# Closed-loop coupling of both physiological spindle model and spinal pathways for sensorimotor control of human center-out reaching

**DOI:** 10.3389/fncom.2025.1575630

**Published:** 2025-08-26

**Authors:** Pablo Filipe Santana Chacon, Isabell Wochner, Maria Hammer, Jochen Martin Eppler, Susanne Kunkel, Syn Schmitt

**Affiliations:** ^1^Institute for Modelling and Simulation of Biomechanical Systems, University of Stuttgart, Stuttgart, Germany; ^2^Hertie Institute for Clinical Brain Research, University of Tübingen, Tübingen, Germany; ^3^Center for Bionic Intelligence Tübingen-Stuttgart (BITS), Tübingen-Stuttgart, Germany; ^4^Faculty of Science and Technology, Norwegian University of Life Sciences, Ås, Norway; ^5^Peter Grünberg Institute (PGI-15), Jülich Research Centre, Jülich, Germany; ^6^Stuttgart Center for Simulation Science, University of Stuttgart, Stuttgart, Germany

**Keywords:** muscle spindle, spinal cord, neuromusculoskeletal model, spiking neural network, proprioception, sensorimotor control, synaptic learning, biomechanics

## Abstract

The development of new studies that consider different structures of the hierarchical sensorimotor control system is essential to enable a more holistic understanding about movement. The incorporation of more biological proprioceptive and neuronal circuit models to muscles can turn neuromusculoskeletal systems more appropriate to investigate and elucidate motor control. Specifically, further studies that consider the closed-loop between proprioception and central nervous system may allow to better understand the yet open question about the importance of afferent feedback for sensorimotor learning and execution in the intact biological system. Therefore, this study aims to investigate the processing of spindle afferent firings by spiking neuronal network and their relevance for sensorimotor control. We integrated our previously published physiological model of the muscle spindle in a biological arm model, corresponding to a musculoskeletal system able to reproduce biological motion inside of the demoa multi-body simulation framework. We coupled this musculoskeletal system to physiologically-motivated neuronal spinal pathways, which were implemented based on literature in the NEST spiking neural network simulator, intended to perform human center-out reaching arising from spinal synaptic learning. As result, the spindle connections to the spinal neurons were strengthened for the more difficult targets (i.e. higher above placed targets) under perturbation, highlighting the importance of spindle proprioception to succeed in more difficult scenarios. Furthermore, an additionally-implemented simpler spinal network (that does not include the pathways with spindle proprioception) presented an inferior performance in the task by not being able to reach all the evaluated targets.

## 1 Introduction

The neural activity that controls motion is complex, even for seemingly simple tasks such as locomotion and reaching movements (Tsianos et al., 2014). In this context, the control of arm and hand movements in humans and non-human primates has fascinated researchers in areas such as psychology, neuroscience and robotics. At first glance, such movements may seem easy to execute, but it is only when attempting to replicate such tasks using artificial systems or when investigating the neural substrate involved in these tasks that we realize the complexity involved (Schaal, 2002). For example, robotic control attempting to reproduce biological reaching movement has to deal with kinematic redundancies, as the same hand position can be reached using different degrees of freedom (DoFs), aligned to different muscle coordination patterns (i.e. muscle redundancy) in humans (Torras, 2002).

Currently still unsolved questions in mammalian motor control remain, which includes regions of cortex but also many extracortical regions: basal ganglia, thalamus, cerebellum, pons, brain stem nuclei and spinal cord (Eccles, 1981; Loeb and Tsianos, 2015). These regions are highly interconnected and each of them has been shown to be important to perform arm reaching in a natural smooth movement (Shadmehr and Wise, 2005; Arber and Costa, 2018).

In this search for a better understanding of sensorimotor control, much has been discussed about the role of spinal cord in voluntary movements, such as reaching and grasping movements performed by the upper limbs. Muscle recruitment has typically been suggested to be programmed and explicitly controlled by signals from various areas of the brain (Hallett et al., 1975; Scheidt and Rymer, 2000; Kargo and Nitz, 2003; Lillicrap and Scott, 2013). However, studies have shown that spinal cord circuits are widely interconnected and can provide more than just reflexive control to muscles (Pierrot-Deseilligny and Burke, 2012). In the modern view, the spinal cord is considered a center for sensorimotor coordination rather than just a relay from the brain to muscles (Raphael et al., 2010). Several descending pathways from the cerebral cortex, cerebellum, vestibular apparatus and brain stem modulate the function of the spinal circuitry rather than directly controlling the activity of muscles responsible for movement (Lavoie et al., 1995; Ivashko et al., 2003). The subcortical structures are the ones responsible for learning the fine-tuning of the motor control (Verduzco-Flores and De Schutter, 2022). Moreover, direct corticomotoneuronal projections are largely absent in opossums, rodents, cats and lower primates, which are still capable of sophisticated limb movements for prey capture and/or food handling (Rathelot and Strick, 2009).

As observed by (Bruel et al. 2024a) in their recent study, the spinal cord also modulates muscle cocontraction (i.e. regulation of simultaneous activation of antagonist muscles), improving the robustness against external motor perturbations; in addition, it has been shown previously that cocontraction can enhance upper limb movement accuracy (Gribble et al., 2003). It was demonstrated that the spinal cord facilitates cerebellar motor adaptation, adding motor control benefits instead of being an evolutionary constraint (Bruel et al., 2024a). Thus, any plausible theory aiming to describe brain function in movement must consider the properties of the spinal circuitry. Besides that, computations in the spinal cord and its synaptic plasticity are still not well-understood. Therefore, the importance of further investigating the contribution of the spinal cord in sensorimotor control, especially in learning of voluntary reaching movements, becomes evident.

In this scenario, computational approaches using modeling and simulation are powerful tools, either for understanding how movement occurs in the biological system, or for using such models as more natural controllers for artificial systems in simulators, robots or prostheses. Computational modeling and simulation of the neuromusculoskeletal system can provide a controlled and predictive environment to study the neuromechanical interaction leading to movement (Klotz et al., 2019). Furthermore, as previously reported (Gizzi et al., 2021a), somatosensory (i.e. afferent) information is elusive and difficult to collect in intact human system; therefore, physiologically coherent neuromusculoskeletal simulations may aid to elucidate the remaining open question of how the central nervous system (CNS) controls the details of movement in a closed-loop manner (Sartori and Sawicki, 2021), together with the development of more efficient human-machine communication and rehabilitation tools. Here, it is relevant to mention the importance of integrating models of the different structures present in the sensorimotor control, in order to verify their interaction and complementarity, enabling a holistic understanding about motion (Edwards, 2010).

Several studies tackled the challenge of modeling the low-level sensorimotor system for voluntary movements in the past. However, they have typically been restricted to one hinge joint operated by a pair of antagonist muscles (e.g., Bullock and Grossberg, 1992; Bashor, 1998; van Heijst et al., 1998; Lan et al., 2005) and usually only incorporated few of the known spinal pathways (e.g., Bullock et al., 1993; Feldman et al., 1998; Maier et al., 2005; Bruel et al., 2024a). Others, more detailed low-level sensorimotor models, incorporate more muscles aligned to a more complete spinal cord circuitry (Raphael et al., 2010; Tsianos et al., 2014). Nevertheless, physiological models of the muscle spindle are rarely incorporated in those detailed low-level models that simulate skeletal muscle mechanics and activation dynamics (Röhrle et al., 2019). Muscle spindle is an essential proprioceptor, playing a crucial role for sensation of limb position and movement in mammals. The main barrier for a broader application of spindle models is that they usually cannot be easily implemented in combination with neuromusculoskeletal models, as detailed in our previous study (Chacon et al., 2023).

We recently developed a physiologically enhanced muscle spindle model (Chacon et al., 2023) that is easily applicable in combination with neuromechanical models, aiming to promote the use of proprioceptive sensors to mimic and understand biological sensorimotor control. Similarly, the use of physiologically-motivated neuromechanical models, that include realistic biomechanical behavior aligned to physiological neuronal circuitry, is essential to allow a more holistic view of the sensorimotor control system. The connection of afferent feedback from proprioceptors to spinal cord neuronal circuitry is a way of integrating different components of the sensorimotor system to understand motor behavior (Haggie et al., 2023), for example, to explore the relationship between spinal connectivity and postural control (Elias et al., 2014). Incorporating more physiologically accurate proprioceptive and neural circuit models into neuromusculoskeletal systems enhances their fidelity, making them better suited for investigating and understanding motor control.

Movement is the result of integration of efferent commands (descending commands) and afferent feedback (including proprioception) (Gizzi et al., 2021a). In experiments where the proprioceptive feedback was stimulated—e.g., the work of (Asín-Prieto et al. 2020) that used skin vibration to enhance motor learning with a robotic ankle exoskeleton—a better performance was observed, suggesting improved motor learning. Thus, further knowledge about proprioception could increase the efficacy of human-machine interface. Furthermore, an understanding of how the execution of upper limb movements occurs in the intact biological system benefits the design of control systems for sensorimotor prostheses (Tsianos et al., 2011). Currently, the main challenge in prosthetic and robotic design is making their movements resemble natural motion (González-Fernández, 2014). Therefore, studies that consider the closed-loop between proprioception and CNS may help to further comprehend the yet open question about the importance of afferent feedback for sensorimotor learning and execution in healthy environment. Consequently, the purpose of this study is to investigate the benefits of the Chacon et al. (2023) spindle model integrated to a biologically-intact neuromusculoskeletal system—able to reproduce biological motion—, connected to a physiologically-motivated neuronal circuitry. This allows us to test whether motor learning and control using a biophysically detailed spinal network—that includes spindle proprioception—is beneficial and results in a more robust performance compared to a simplified spinal cord network.

A spinal cord circuitry model was reproduced and adapted from literature (Raphael et al., 2010; Tsianos et al., 2014), which considers patterns of change in muscle regulation necessary to control multiple muscles and DoFs, aligned with a variety of complex sensorimotor behaviors. In this spinal cord model, the structure and properties of known spinal pathways were taken in consideration (Pierrot-Deseilligny and Burke, 2005) by implementing different interneurons aligned to the connectivity that they establish between afferents from a specific muscle and motoneuron of the same muscle (homonymous), synergistic muscles and antagonistic muscles. Therefore, the novelty of our work is that we developed a simulation environment that incorporates a physiologically enhanced muscle spindle model in a neuromechanical arm model, together with the closed-loop coupling to a detailed spinal cord model. This was combined with a deliberately simple brain model in order to force the spinal circuitry to generate all the required dynamics and investigate its interaction with the spindle afferent firings to succeed during reaching task.

Additionally, this study novelly implements the connection of two independent softwares for modeling and simulation: a multi-body simulation framework [demoa (Schmitt, 2022)] and a spiking neural network simulator [NEST (Haug et al., 2023)]. This creates a powerful simulation environment that allows modeling and communication of musculoskeletal models with spiking neural networks, as each software represents a detailed open-source simulation tool in its respective field (the work has been made freely available as described in Data Availability Statement). This shall benefit future work by motivating the development of further neuromusculoskeletal models in such environment, which will boost the understanding of sensorimotor control.

## 2 Materials and methods

In this section, we describe the technical connection between the biomechanical framework and the spiking neural framework (Section 2.1), followed by the details of the spinal cord network and how afferent firings of the spindle are processed (Section 2.2). Finally, we present the sensorimotor task performed to verify the orchestration between spindle and spinal cord models, and we describe the learning process represented by the optimization of spinal synaptic weights (Section 2.3).

### 2.1 Connection of biomechanical framework with spiking neural framework

#### 2.1.1 Musculoskeletal model in demoa

The muscle spindle model of (Chacon et al. 2023), representing the intrafusal fibers, was implemented inside the demoa simulation framework (Schmitt, 2022), in order to allow its integration in other muscle-driven models previously developed within the framework. Demoa is a biophysics simulator for muscle-driven motion. It has been developed with the intention to model and simulate 3D biomechanical problems, using standard solvers known to work for systems of ordinary differential equations. The simulator is actively maintained by the Computational Biophysics and Biorobotics (CBB) group of the University of Stuttgart, and it is available as open source code.^1^

**Figure 1:**
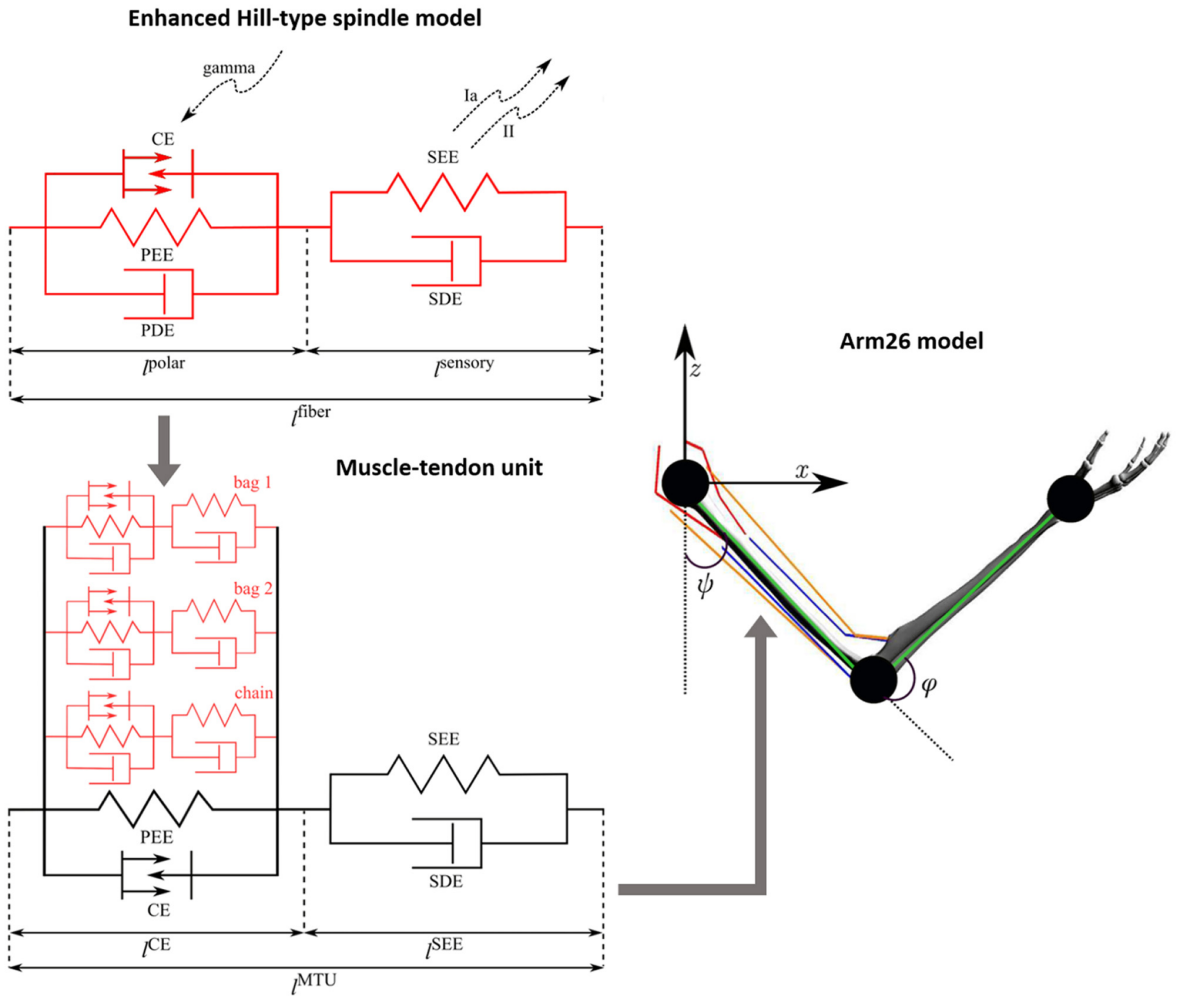
Schematic of the Arm26 model (Wochner and Schmitt, 2022) with the enhanced Hill-type spindle model (Chacon et al., 2023) integrated to its muscle-tendon units (MTUs) (Haeufle et al., 2014). The mechanical components correspond to contractile element (CE), parallel elastic element (PEE), serial elastic element (SEE), parallel damping element (PDE), and serial damping element (SDE). The spindle fibers include gamma activation on the polar region (*l*^*polar*^) and afferent firings (Ia and II) from the sensory region (*l*^*sensory*^). The full spindle model comprises three different fiber types (bag 1, bag 2, and chain) with qualitatively identical mechanical components, coupled in parallel to the belly part of the MTU (i.e. parallel to CE and PEE). The Arm26 model has two joints and the joint angles ψ (shoulder) and φ (elbow), including six MTUs: two monoarticular shoulder muscles (red lines), two monoarticular elbow muscles (blue lines) and two biarticular muscles (orange lines). The kinematic chain (green lines) of the arm model is also showed.

The muscle model, used in this study, is identical with the Hill-type model of (Haeufle et al. 2014), representing the extrafusal fibers. The integration of the spindle model (intrafusal fibers) in the muscle model (extrafusal fibers) was done by modeling the intrafusal fibers of the spindle model in parallel to the active part of the extrafusal fibers (Chacon et al., 2023). The muscle spindle model (intrafusal fibers) is formed by three different fiber types (bag 1, bag 2, and chain), and thus, each of these fibers have been implemented in parallel to the muscle belly of the extrafusal fibers, just like in biology. Figure 1 depicts the new, combined extrafusal and intrafusal fiber model that is now available in demoa.

In the present work, we use a modified version of the neuromusculoskeletal model Arm26 (Wochner and Schmitt, 2022). This model comprises a musculoskeletal model of the arm with two DoFs actuated by six muscles, combined with a simple open loop controller of the muscles. The geometry and muscle parameters are derived from previous studies (Bayer et al., 2017; Driess et al., 2018). For a complete list of parameters, see Wochner and Schmitt (2022). The model has two rigid bodies (lower and upper arm) that are connected via two one-degree-of-freedom revolute joints that refer to the shoulder and elbow joints. The model's actuation arises from active forces generated by six muscle-tendon units (MTUs, Haeufle et al., 2014) [four monoarticular muscles—shoulder anteversion (MSA), shoulder retroversion (MSR), elbow flexor (MEF), elbow extensor (MEE)—and two biarticular muscles—biarticular flexor (BEFSA), biarticular extensor (BEESR)]. The Arm26 model provides the necessary level of biomechanical detail to determine internal muscular and joint loads as well as muscle-bone contact forces, being successfully used in former works to perform reaching task and predict personalized forces for assistive devices, for example (Wochner et al., 2020; Stollenmaier et al., 2020). In the present work, all six MTUs of the Arm26 model have been replaced by the new, combined muscle and spindle model, described above (Figure 1).

#### 2.1.2 NEST

NEST^2^ is an open source simulation engine that has evolved with the neuroscience community over a quarter-century (Gewaltig and Diesmann, 2007). The simulator has been continuously advanced and extended for large-scale brain simulations at the resolution of single neurons and synapses. The code is scalable (Jordan et al., 2018), where recent technological advancements also target GPU-based systems (Golosio et al., 2023). Neuron models in NEST range from simple point-neuron models with linear subthreshold dynamics, which can be integrated exactly, over more complex point-neuron models that require numerical integration, to multi-compartment models that allow investigating dendritic effects (Rotter and Diesmann, 1999). Synapses are static in the simplest case meaning that synaptic weight and transmission delay are fixed. NEST uses hybrid parallelization typically running one MPI process per compute node or socket with frequent collective spike communication combined with multi-threading (Morrison et al., 2005; Plesser et al., 2007). The simulation advances in a globally time-driven scheme, where the minimum synaptic transmission delay in the network defines the spike-communication interval, but independently, neurons can be updated on a finer time grid (Morrison and Diesmann, 2008).

The elements of a connection in NEST are: sending node, receiving node, weight, and delay. The weight corresponds to the synaptic weight, determining how strong the connection is and consequently intensifying or inhibiting the signal between the nodes. Analogously, the delay corresponds to the synaptic delay, specifying the time to the signal to be transmitted from the sending node to the receiving node. This signal is represented as event in NEST. Each event contains the time of its creation and the connection weight. For correct timing, a transmission delay is added to the time the event is created to account for the propagation time to the postsynaptic neuron (or node). The effect of the event on the post-synaptic neuron depends on the synapse model, with the simplest one delivering the exact connection weight. Furthermore, NEST has different event types, which depend on the type of extra information that the event is carrying, for example, a *SpikeEvent* carries the spike times (see Gewaltig and Diesmann, 2007 for further details).

#### 2.1.3 Coupling of demoa and NEST

The coupling of the simulator frameworks demoa and NEST requires a precise synchronization of the two. Therefore, it is crucial that the two simulators run in lockstep mode, i.e., they start synchronously and run in parallel, while exchanging their time-critical information periodically. In particular, NEST sends the spike times originating from the motoneurons in the spinal cord network to demoa, which are then converted in spike trains to stimulate the muscle fibers. In the other direction, demoa needs to send afferent firing rates to NEST, reflecting changes inside the neuronal network.

The two simulators use different interfaces to control the simulation experiments (demoa is written in C/C++ code, NEST is controlled using Python and PyNEST scripts). For the purpose of this study, a time-efficient approach was created by extending demoa with a Python interface, to use Python as common programming language for the coupled framework. The interface is implemented on top of *Cython*, an extension for Python language that allows to link Python code directly to C code and achieve good runtime performance (Behnel et al., 2011). As communication protocol to exchange data between the two coupled simulators, we use *dictionaries*. Dictionaries are associative arrays that map entity names to their respective values. While NEST already used dictionaries in its user interface layer, demoa has been extended to provide this feature as well.

Concretely, NEST sends times of spikes originating from the motoneurons in the spinal cord network, which then generate spike signals at the given time stamps in demoa. A spike signal in demoa is modeled as a normalized exponential curve, going from *zero* to *one* value–according to the all-or-nothing principle of neuronal firing (Kandel et al., 2013)—and decaying exponentially according to the equation below:


(1) $$\begin{array}{l} u_{t}=u_{t-1}\times e^{\frac{-1}{\tau }\times \left(t-t_{old}\right)}, \end{array}$$

where *u*_*t*_ refers to the fiber stimulation represented in the form of a spike in that case; *u*_*t*−1_ is the fiber stimulation before the new spike time has arrived; *t* is the spike time just received (also the current simulation time); *t*_*old*_ is the previous spike time received (in case, a spike has happened before); τ = 0.01*s*. The spike signals generated in demoa are passed as fiber stimulation (*u*) to both extrafusal fibers (the muscle or MTU) and intrafusal fibers (the spindle), as α and γ stimulations in order to reproduce the Rockenfeller and Günther (2018) activation dynamics in the muscle and spindle fibers.

**Figure 2:**
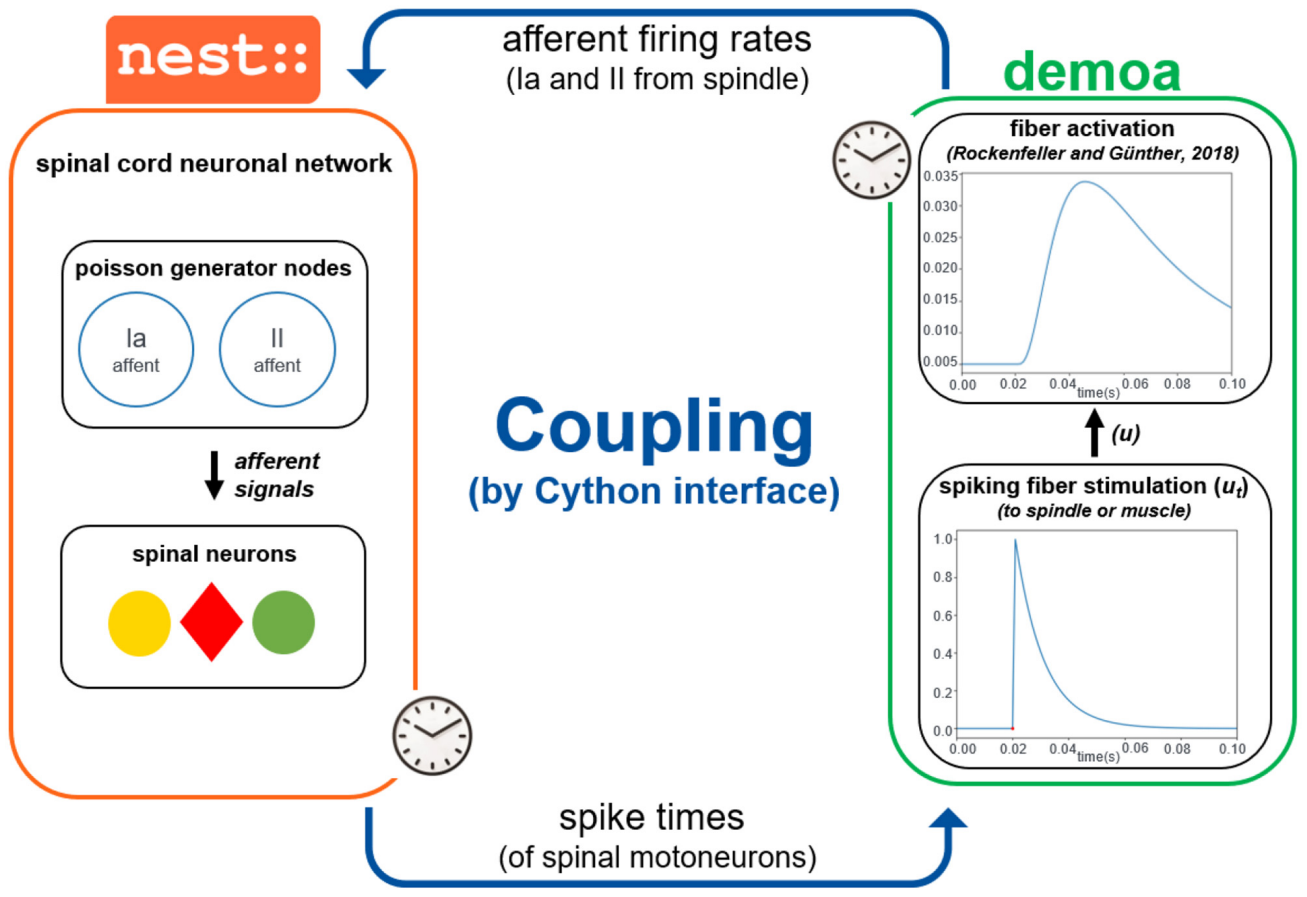
Coupling of demoa and NEST simulators. With both simulators starting at same time and running in parallel, they exchange information in a time-critical way by a Cython interface: NEST sends spike times of spinal motoneurons to demoa, and demoa sends spindle Ia and II afferent firing rates to NEST. Demoa receives only the spike times from NEST and it generates a spike signal that happens in the spike time received (according to the Equation 1), corresponding to the spiking stimulation (*u*_*t*_) to the spindle or muscle fibers. The *u*_*t*_ is passed as the fiber stimulation (*u*) to reproduce the Rockenfeller and Günther (2018) activation dynamics in the spindle and muscle fibers. Simultaneously, demoa sends the afferent firing rates generated from the spindle model to the spinal cord network in NEST, which adjusts the firing rate of the poisson generator nodes in the network (separated nodes for Ia and II afferents). The afferent signals from the poisson generators project to the corresponding spinal neurons.

The information sent from demoa to NEST are the afferent firings (Ia and II afferents) from the muscle spindle model. These afferent firings are represented in the form of a frequency of spikes (pulses per second). The frequencies are passed to NEST to update the firing frequency of the nodes in the spinal cord network. NEST permits the creation of *poisson generators*, which are nodes in the network that simulate neuron firing with Poisson statistics, i.e. exponentially distributed interspike intervals. These nodes can have their firing rate updated (in spikes per second). Therefore, poisson generators were created to implement afferent firings inside the NEST network, which have their rate adjusted based on the afferent firing rates received from demoa. The structure of the spinal cord network is described in details in the next section. See Figure 2 for a summary of the coupling between the simulators.

### 2.2 Processing of spindle afferents by spinal cord network

We developed a spinal cord model in NEST inspired by the spinal circuitry previously implemented by Raphael et al. (2010) and Tsianos et al. (2014), but with modifications. The previous work extracted structure and properties of known spinal pathways from literature (Pierrot-Deseilligny and Burke, 2005). They implemented four classic types of interneurons (Ia inhibitory, Ib inhibitory, propriospinal and Renshaw), and a connectivity of afferents from a specific muscle and the alpha motoneuron of the same muscle (homonymous), synergistic muscles and antagonistic muscles. Thus, five classic spinal pathways were reproduced:

1. stretch reflex pathway,
2. propriospinal pathway,
3. inhibitory Ia pathway,
4. inhibitory Renshaw pathway and
5. inhibitory Ib pathway.

**Figure 3:**
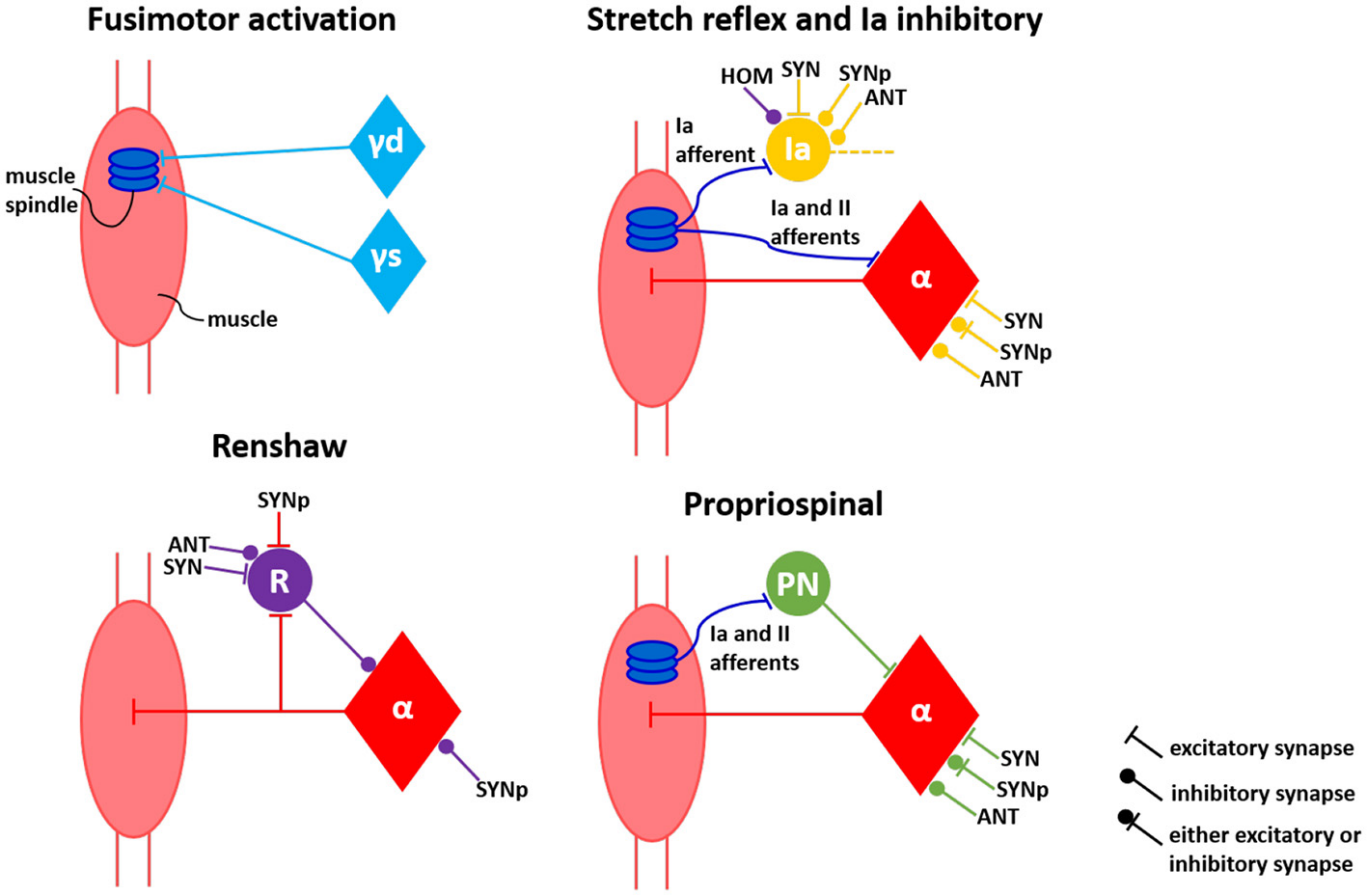
Connectivity model of spinal circuitry. Fusimotor activation aligned to four classical interneuronal pathways—stretch reflex, inhibitory Ia, inhibitory Renshaw and propriospinal—are represented by the perspective of a single muscle. Neuronal connections from self (HOM), antagonist (ANT), synergist (SYN) and partial synergist (SYNp) muscles are displayed. γd, γs, α, Ia, R, and PN correspond to dynamic gamma, static gamma and alpha motoneurons, together with Ia, Renshaw and propriospinal interneurons, respectively.

The stretch reflex pathway directly excites the alpha motoneuron, while the other pathways influence the motoneuron activity through the respective interneuron. The modifications in the present work concerns the Golgi Tendon Organ (GTO), which we did not implement. Thus, we did not include the inhibitory Ib pathway to the circuitry, because the main afferent signal that projects to the Ib interneurons comes from GTO. The propriospinal pathway was still implemented, but with the propriospinal interneurons receiving only the spindle afferents. Additionally, in our work there is a clear distinction between primary (Ia) and secondary (II) afferents coming from the muscle spindle model, this allows to direct each of these afferents to the specific neuronal components innervated by the Ia and II fibers. The alpha motoneurons and the propriospinal interneurons receive both Ia and II afferents in the stretch reflex and propriospinal pathways, respectively. On the other hand, the Ia interneurons receive only the Ia afferent from spindle in the Ia inhibitory pathway. As a further physiological enhancement compared to the previsou work of Raphael et al. (2010) and Tsianos et al. (2014), we explicitly included gamma motoneurons to the spinal circuitry and their fusimotor activation. This implementation allows for two independent pools of neurons representing the dynamic and the static gamma motoneurons (with the dynamic gamma motoneurons only innervating the bag 1 of the spindle and the static gamma motoneurons innervating bag 2 and chain). Therefore, eventually four spinal pathways were reproduced—stretch reflex, propriospinal, inhibitory Ia and inhibitory Renshaw pathways—differentiating between Ia and II fibers' projection, aligned to fusimotor activation represented by distinct pools of dynamic and static gamma motoneurons (see Figure 3).

In order to reproduce the spinal pathways and respective connections, we implemented six different pools of neurons in NEST:

1. alpha motoneurons,
2. dynamic gamma motoneurons,
3. static gamma motoneurons,
4. Ia interneurons,
5. Renshaw interneurons and
6. propriospinal interneurons.

Each MTU of the Arm26 model contains a small network defined by one neuron of each neuronal type, comprising six neurons per muscle representing the pools of neurons directly effecting such muscle. As the Arm26 model has six MTUs, the spinal cord model implemented in NEST comprises on total 36 neurons. Each of these neurons was created with the *af_psc_alpha* model from NEST. Such neuronal model corresponds to a leaky integrate-and-fire neuron with alpha-shaped synaptic input currents that has separate excitatory and inhibitory components (Gewaltig and Diesmann, 2007). The distinction between excitatory and inhibitory synapses is important for defining the relations between neuronal components of different muscles in the current work, as detailed below. Also, the neuronal model allows a constant input current set as a model parameter during the creation of the neuron. This way, we could use this input current to model the supraspinal control projecting to the spinal neurons (descending commands). As observed in previous work (Verduzco-Flores and De Schutter, 2022), only the supraspinal projection to the interneurons was enough to succeed during a reaching task, however, they still introduced direct connections from primary motor cortex to motoneurons because in higher primates these connections are present for distal joints (Lemon, 2008). Therefore, in our work, all the motoneurons and interneurons of the spinal network received a current input modeling the convergent inputs from primary motor cortex. The value of such current was defined as 380 pA for all neurons, as we observed that this value led to a prior mid-level activation of the muscle fibers, supporting further optimizations.

The muscles around the joints can enter into various functional relationships with each other, which can be antagonistic, synergistic, or partially synergistic. These functional relations influence the connectivity between the neurons of the involved muscles. In a given movement, the muscles involved in that movement are called synergists (or agonists), while those performing the opposite movement are called antagonists (Calais-Germain, 2002). Synergist and antagonist projections are such that a signal from a given muscle has the same effect on synergist muscles and the opposite effect on antagonist muscles. The synergist-antagonist arrangement is commonly observed in the spinal cord (Pierrot-Deseilligny and Burke, 2005). Pairs of muscles identified as partial synergists can behave either as antagonists or synergists depending on the sensorimotor task, i.e. the antagonist or synergist relation is not strongly defined in that case. The pair of muscles in the Arm26 model defined as antagonists, synergists and partial synergists are listed in the Table 1.

**Table 1:** Relations defined between pair of muscles in the Arm26 model.

**Muscle pair**	**Relation**
MEF–MEE	Antagonists
BEFSA–BEESR	Antagonists
MSA–MSR	Antagonists
BEFSA–MEF	Synergists
BEFSA–MSA	Synergists
BEESR–MEE	Synergists
BEESR–MSR	Synergists
MEF–MSA	Partial synergists
MEF–MSR	Partial synergists
MEE–MSA	Partial synergists
MEE–MSR	Partial synergists

The muscles correspond to: muscle elbow flexor (MEF), muscle elbow extensor (MEE), muscle biarticular flexor (BEFSA), muscle biarticular extensor (BEESR), muscle shoulder anteversion (MSA), muscle shoulder retroversion (MSR).

The connections between neurons from different muscles depending on their relations are also depicted in Figure 3, based on literature (Pierrot-Deseilligny and Burke, 2005; Raphael et al., 2010; Tsianos et al., 2014). The connections between the neurons of synergist muscle pairs are the opposite of those between antagonist muscle pairs; in other words, if a particular connection between neurons of synergist muscles is excitatory, this connection is inhibitory between antagonist muscles. The excitatory synapses were defined in NEST as receiving an initial synaptic weight of 5.0, whilst the inhibitory synapses was initialized with a synaptic weight of –5.0. In addition, some connections between partial synergist muscles can be either excitatory or inhibitory, therefore, these connections initially received a synaptic weight of 2.0, enabling it later to turn into negative (inhibitory) synapses or staying as positive (excitatory) synapses during the subsequent optimization process of sensorimotor task learning, as described in the next section. All the connections in the spinal cord network were implemented with a 1-ms physiological synaptic delay.

### 2.3 Learning of sensorimotor task

**Figure 4:**
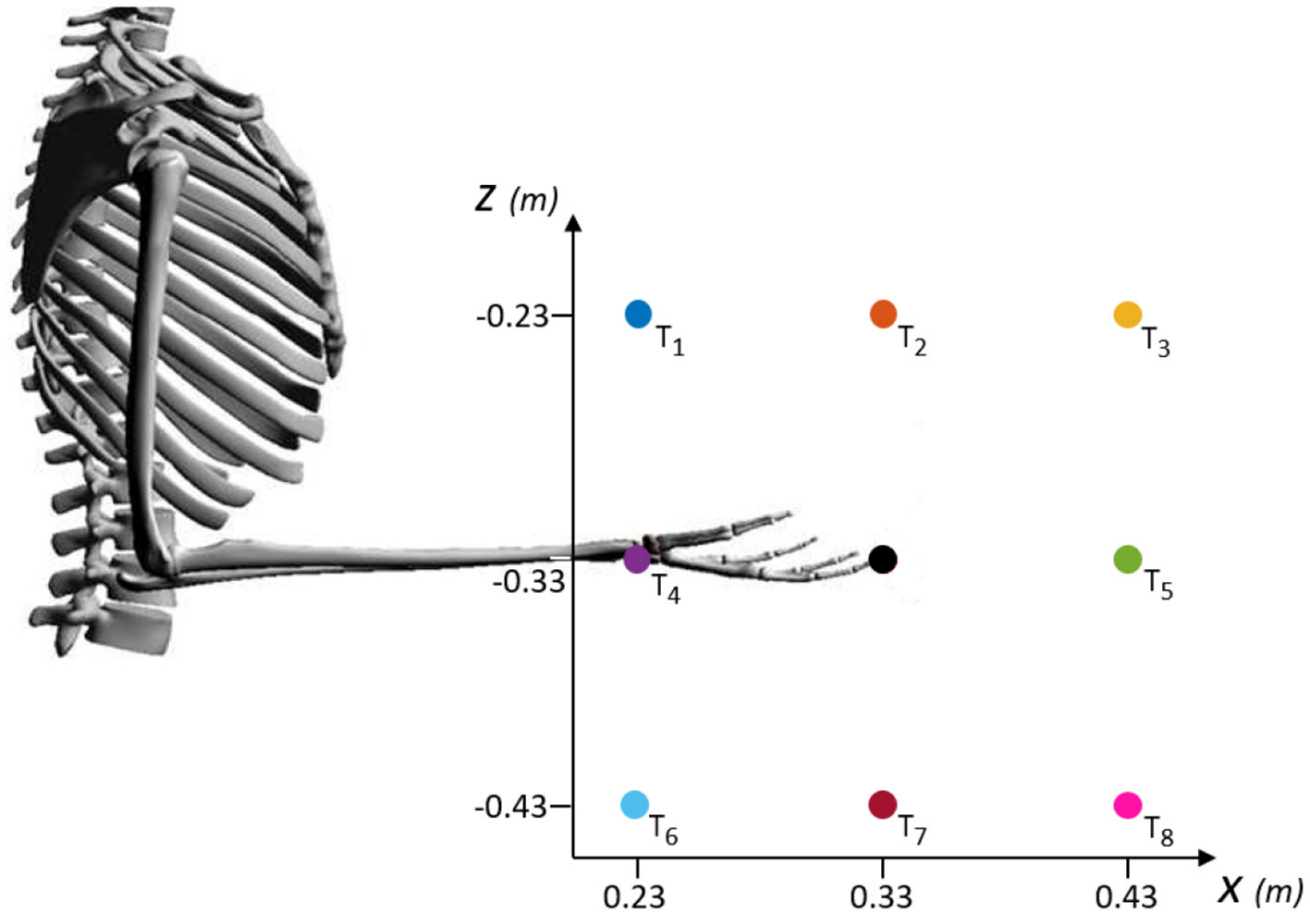
Schematic of the targets evaluated during center-out reaching task in the *XZ*-plane. Eight peripheral targets (*T*_1_ to *T*_8_) spaced in a 0.1m-distance around the coordinates of an initial hand position ([0.33, –0.33] m, black dot). The initial position corresponds to an arm posture of 90° for the elbow angle and 0° for the shoulder angle.

For the analysis of the sensorimotor control in our neuromusculoskeletal environment—formed from muscle spindle to spinal circuitry, connecting two different biological simulators—, we chose the intensely studied point-to-point reaching task. Performance of an upper limb model during planar (2D) reaching has been extensively evaluated in previous studies (Kistemaker et al., 2014; Dura-Bernal et al., 2015; Driess et al., 2019; Wochner et al., 2020), including work that inspired our spinal circuitry model (Tsianos et al., 2014), and more recent works which implemented spinal cord models as well (Verduzco-Flores and De Schutter, 2022; Bruel et al., 2024a). In this study, we specifically used a standard center-out reaching task to assess our modeling approach in reaching desired hand positions for several directions from scratch: eight peripheral targets around an initial hand position, with a 0.1m-distance between their coordinates (see Figure 4).

The simulation time was set to 0.8s, similar to (Wochner et al. 2022), who compared the timing with experimental data of human point-reaching movement. The simulation started from the [0.33, −0.33]m-central-position, corresponding to an arm posture of φ = 90° for the elbow angle and ψ = 0° for the shoulder angle. This initial condition can be seen in Figure 4 and the angles are defined in Figure 1.

From the simulators' perspective, demoa and NEST are started at the same time and both are finished 0.8s later. During this time frame, the simulators exchange information every 0.1s, i.e., eight times during simulation. Specifically, NEST sends the spike times of alpha, dynamic gamma and static gamma motoneurons to demoa, and demoa sends the primary and secondary afferent firing rates to NEST, as previously described. The model learned to reach one target at a time, following the optimization workflow detailed below.

#### 2.3.1 Optimization of spinal synaptic weights

The learning process in the neuromusculoskeletal model to perform the center-out reaching task was implemented by optimization of the synaptic weights present in the neuronal connections of the spinal circuitry model, representing physiological neuronal learning. For each of the eight targets, the weights were optimized from their initial values (5.0 for excitatory synapses and –5.0 for inhibitory synapses) within a pre-defined range (0.0 to 10.0 for excitatory synapses and –10.0 to 0.0 for inhibitory synapses). Also, the specific connections between partial synergist muscles, that can be either excitatory or inhibitory, had their weights optimized from the initial value (2.0) and their optimization range allowed for them to be either excitatory or inhibitory synapses (–10.0 to 10.0).

As learning algorithm, we used the covariance matrix adaptation evolution strategy (CMA-ES) algorithm, from the implementation available at Python Package Index (PyPi) (Hansen et al., 2023). CMA-ES is a stochastic method for real-parameter (continuous domain) optimization of non-linear, non-convex functions, belonging to the class of evolutionary algorithms and evolutionary computation (Hansen, 2016). This optimizer is especially useful for difficult high-dimensional optimization problems in continuous search spaces.

In each iteration of the algorithm, the 0.8s-simulation of center-out reaching was performed for the individual assessed target. Every simulation starts from the same given set point with the hand at the initial shown position in Figure 4.

The number of synaptic weights optimized in the spinal cord circuitry was 150, corresponding to the total neuronal connections in the network. The algorithm had a maximum iteration number of 1,000 and a population size of 150, matching the number of weights. Usually, to prevent the population degeneration into a subspace, search algorithms require a large population size (considerably larger than the problem dimension). However, in CMA-ES, the population size can be freely chosen, because its learning rates prevent the degeneration even for small population sizes (Hansen, 2016). Small population sizes normally lead to faster convergence, thus, aiming to reduce the optimization time for the already large optimization problem, we decided for a population size that coincides to the problem dimension.

As cost function, we used the Euclidean distance to the desired target, in two different forms. First, at the start of optimization process, the cost function calculates the sum of Euclidean distances between all points of the simulated hand-trajectory to the target. This function aims to get the trajectory close to target since the simulation starts, leading to a faster reaching with a more direct trajectory. Then, when the algorithm solution finally leads to trajectories that terminate closer to the target (specifically, with a difference < 0.05m between the Cartesian coordinates of the final hand position and the target), the cost function changes to the Euclidean distance only between the final point of simulated trajectory and the target. This change forces the final hand coordinates to coincide with the target coordinates at the simulation end. Thereby, the adopted cost function corresponds to the following formulation: if ||*x*_*end*_−*x*_*ref*_|| < 0.05*m* and ||*z*_*end*_−*z*_*ref*_|| < 0.05*m*:


(2) $$\begin{array}{l} J={\left(x_{end}-x_{ref}\right)}^{2}+{\left(z_{end}-z_{ref}\right)}^{2}, \end{array}$$

else:


(3) $$\begin{array}{l} J=\overset{n}{\underset{i=1}{\sum }}{\left(x_{i}-x_{ref}\right)}^{2}+{\left(z_{i}-z_{ref}\right)}^{2}, \end{array}$$

where *x*_*end*_ and *z*_*end*_ correspond to the respective coordinates of end hand position, and *x*_*ref*_ and *z*_*ref*_ correspond to the respective coordinates of desired target (reference coordinates). *n* corresponds to the number of points in the simulated trajectory.

The neuromusculoskeletal model was optimized individually for the eight targets, following the optimization setup presented above, with the original segments' mass of the Arm26 model: 2.10 kg for the upper arm and 1.65 kg for the lower arm (Wochner and Schmitt, 2022). To introduce a perturbation into the system, we increased the mass of the distal (lower) arm segment by 1 kg. This added mass represents a dynamic load, simulating a real-world task such as holding or lifting an object (e.g., a bottle of water). Since the model does not explicitly simulate the wrist or hand, the added mass is applied directly to the forearm, effectively increasing the inertial and gravitational load that the muscles must counteract. This perturbation necessitates changes in muscle activation patterns—particularly requiring increased activation of the elbow flexors to achieve the same reaching trajectory.

Furthermore, in order to verify the importance of the proprioception feedback to the neuromusculoskeletal model—represented by the processing of primary and secondary afferent firings from muscle spindle model by spinal circuitry model—, we implemented a simpler spinal cord model, removing the spinal pathways that depend on the spindle afferents. Using this approach, we can observe the behavior of a spinal circuitry that does not consider proprioception, also corresponding to a minimization of neuronal pathways for comparison to a more complete implementation—as similarly performed in previous studies like (Bruel et al. 2024b). Therefore, from the pathways depicted in Figure 3, only the Renshaw pathway was implemented in the new spinal circuitry. The synaptic weights of this simpler network were also optimized to perform the center-out reaching task, with the same optimization setup as described for the previous scenarios, including the perturbed training (mass increment of lower arm segment). The simpler spinal network contains only 42 synaptic weights which need to be optimized. To maintain a consistent optimization configuration over all the evaluated scenarios, the optimization of the simpler spinal circuitry also had a population size of 150, together with a maximum iteration number of 1,000.

In summary, the synaptic weights within spinal circuitry were optimized for the center-our reaching task in four different scenarios, as listed in Table 2.

**Table 2:** Scenarios where the spinal synaptic weights were optimized to perform center-our reaching task.

1	Complete spinal circuitry model (including spinal pathways with spindle afferents) without perturbation (original mass of Arm26 segments)
2	Complete spinal circuitry model with perturbation (increment of lower arm segment in 1 kg)
3	Simpler spinal circuitry model (without spindle proprioception—only Renshaw pathway) without perturbation
4	Simpler spinal circuitry model with perturbation

## 3 Results

### 3.1 Neuronal firing from spinal cord to muscle and spindle responses

Here we demonstrate the proof-of-principle study of the coupling between two simulators, demoa and NEST, exhibiting the spiking firing from the motoneurons (alpha and dynamic/static gammas) in the spinal cord model implemented in NEST, which is translated to muscle stimulation and fusimotor drive (spindle stimulation) in demoa, reflected in muscle activation and spindle afferent firings, respectively.

**Figure 5:**
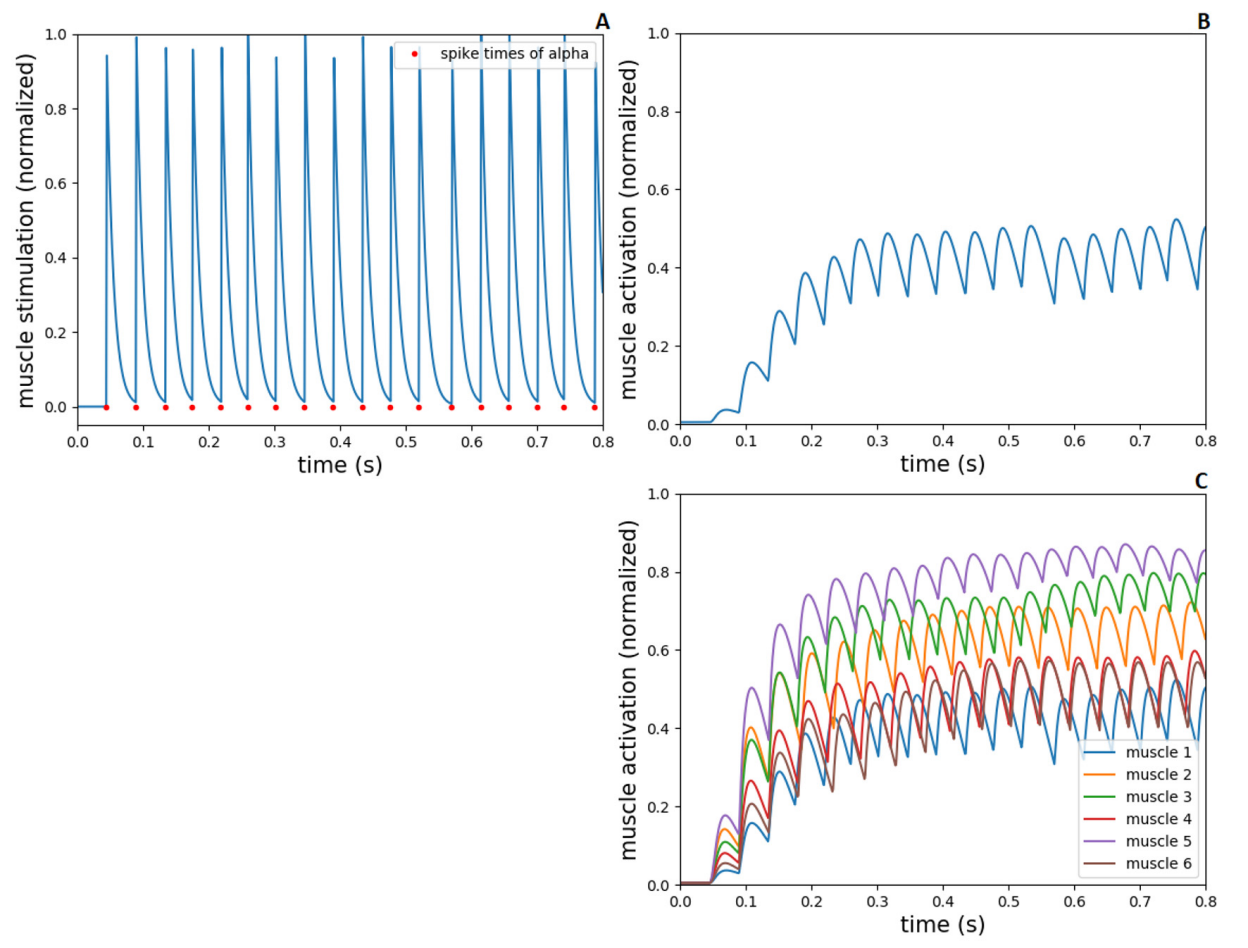
Spiking stimulation from alpha to muscle activation, during 0.8s-simulation in NEST and demoa, without muscle pre-activation. **(A)** Spike times of alpha motoneuron from NEST and conversion to spiking muscle stimulation in demoa, for the muscle 1. **(B)** Muscle activation in demoa resulted from spiking stimulation for the muscle 1 in **(A)**. **(C)** Muscle activation for the six muscles of Arm26 model. Muscles 1 to 6 correspond to muscle elbow flexor (MEF), muscle elbow extensor (MEE), muscle biarticular flexor (BEFSA), muscle biarticular extensor (BEESR), muscle shoulder anteversion (MSA) and muscle shoulder retroversion (MSR), respectively.

The alpha motoneurons innervate muscle extrafusal fibers, therefore, the spike times of alpha neurons from the spinal cord network were converted to spiking muscle stimulation (*u*_*t*_) in demoa (according to Equation 1), resulting in muscle activation which is calculated by the (Rockenfeller and Günther 2018) activation dynamics. In Figure 5, we present the muscle stimulation and activation during a 0.8s-simulation run. The end time of the simulation has been adjusted to the execution time of this task found in human experiments (see Section 3.2.1). For the plots, no pre-activation of the muscle fibers was introduced, aiming to exhibit only the effect of the spiking muscle stimulation to the muscle activation. The synaptic weights in spinal connections have fixed initial values (5.0, –5.0 and 2.0), and the input current to the spinal neurons is set to 380 pA, as explained in Section 2.2.

Figures 5A, B shows the spiking muscle stimulation and muscle activation, respectively, exemplary for the muscle 1 (MEF). In Figure 5C, the muscle activation for all the muscles are presented. As displayed, the value of neuronal input current (modeling the supraspinal control projecting to the spinal neurons, i.e descending commands) results in an initial spiking firing that leads to a mid-level activation of muscle fiber (around 0.5 of normalized value between 0 and 1), supporting the further optimizations to accomplish sensorimotor learning.

**Figure 6:**
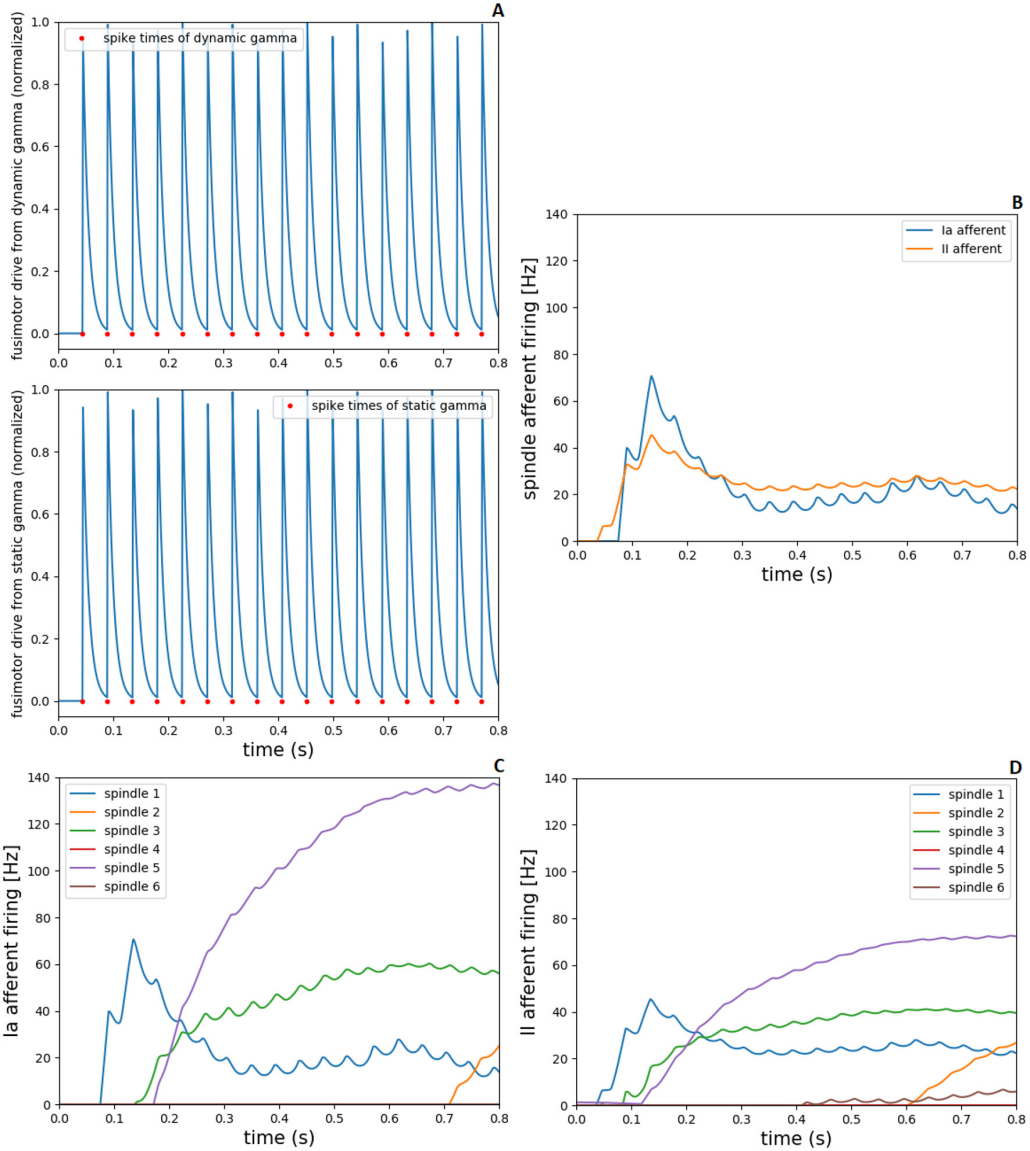
Fusimotor drive from dynamic and static gammas to spindle afferent responses, during 0.8s-simulation in NEST and demoa, without muscle pre-activation. **(A)** Spike times of dynamic and static gamma motoneurons from NEST and conversion to spiking fusimotor drive in demoa, for the spindle in muscle 1. **(B)** Ia and II afferent firings from muscle spindle model in demoa during fusimotor drives depicted in **(A)**, also for the spindle in muscle 1. **(C, D)** Ia and II afferent firings for the six spindles implemented in Arm26 model. Spindles 1 to 6 correspond to the respective spindles implemented in muscles 1 to 6 of Figure 5.

The gamma motoneurons innervate spindle intrafusal fibers (dynamic gamma to bag 1 fibers and static gamma to bag 2 and chain fibers), therefore, the spike times of gamma neurons from spinal cord model were converted to spiking fusimotor drive in demoa, alike the implementation for alpha neurons. This fusimotor drive is used to calculate the spindle activation, similar to the muscle activation by (Rockenfeller and Günther 2018) activation dynamics, reflecting in modulation of Ia and II afferent firings from the muscle spindle model. in Figure 6, we present the fusimotor drive from dynamic and static gammas, together with Ia and II afferent firings from spindle model, during the same 0.8s-simulation as in Figure 5.

Figure 6A depicts the spiking fusimotor drive from both gamma types, for the spindle implemented in muscle 1 (MEF). In Figure 6B, the afferent firings from the same spindle of A are shown. Figures 6C, D shows the Ia and II afferent firings, respectively, from the spindles of all muscles in the arm model. It is observed that although there is activation in all muscles, some spindles presented zero or low afferent firings during the simulation [spindles of muscles 4 (BEESR) and 6 (MSR)]. This happens because the muscle activation does not necessarily lead to changes in muscle lengths (isometric contraction), and the spindle fires proportionally to the muscle fiber length change and rate of length change. Thus, during the depicted simulation, muscles 4 and 6 did not present a length change that reflected in high afferent response from the spindle model attached to them.

### 3.2 Performance of sensorimotor task after optimization

To assess the performance of this proof-of-principle study, we simulated reaching tasks and qualitatively compared the results with previous works from literature of both experimental recordings and computer simulation studies.

#### 3.2.1 Reaching hand-trajectories

Regarding the analysis of reaching hand-trajectories subsequent to optimization step, the performance of simulated trajectories was evaluated based on distance to the reference targets at the end, i.e. whether the arm model was able to reach the evaluated targets after completion of learning (optimization) process in the spinal cord model.

**Figure 7:**
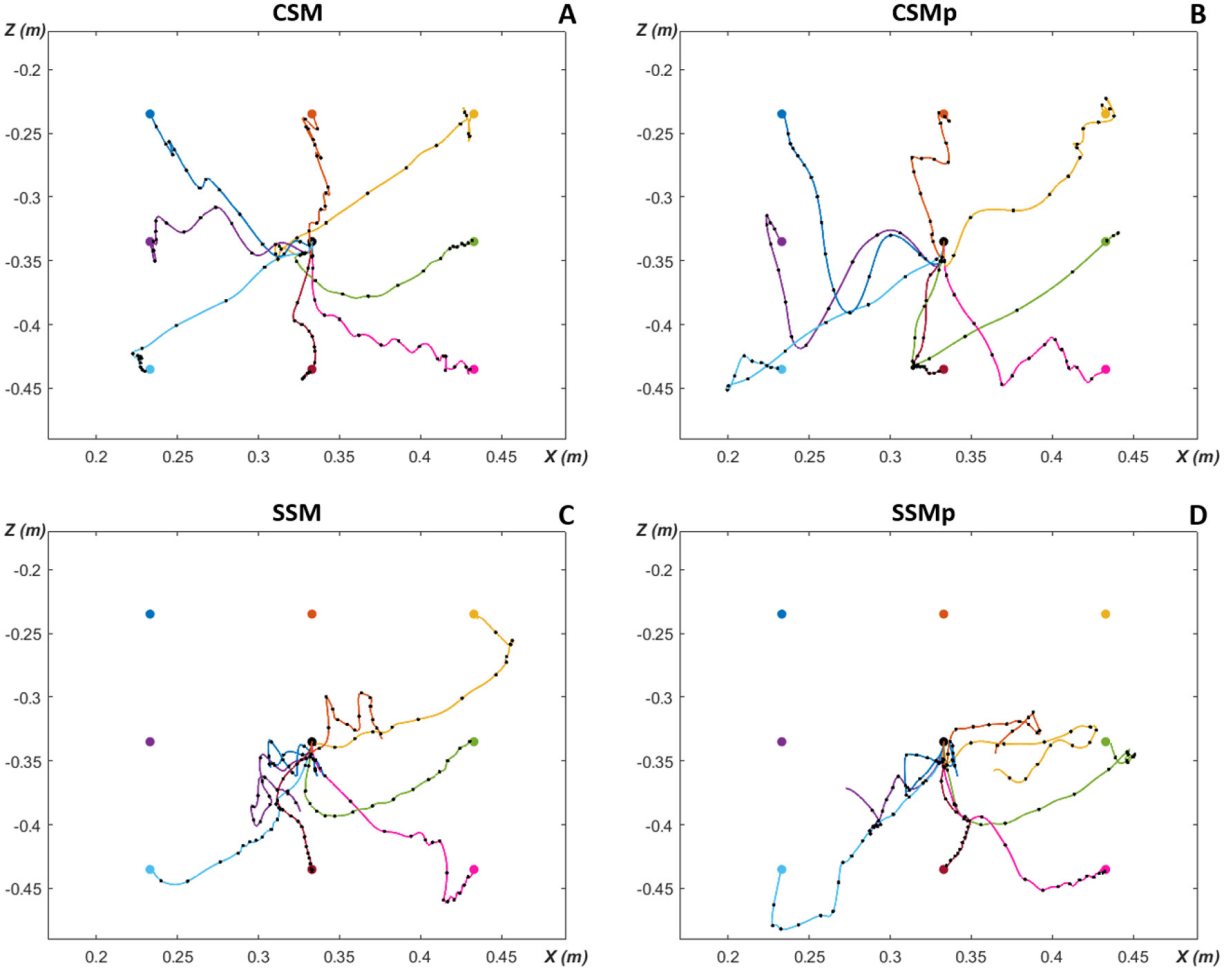
Planar reaching hand-trajectories with timing characteristics, after optimization for the eight evaluated targets around the initial position. Overlaid black dots on reaching trajectories are spaced 50 ms apart to illustrate the timing characteristics of the movement. **(A)** CSM, complete spinal circuitry model without perturbation. **(B)** CSMp, complete spinal circuitry model with perturbation (increment of lower arm segment in 1 kg). **(C)** SSM, simpler spinal circuitry model (without spindle proprioception—only Renshaw pathway) without perturbation. **(D)** SSMp, simpler spinal circuitry model with perturbation. The color of trajectory line to each target matches the target color.

In Figure 7, we show the post-optimization trajectories for the eight targets in the *XZ*-plane of hand movement, for the four evaluated scenarios in the neuromusculoskeletal model. The eight targets were reached in the first scenario with the complete spinal cord network and original mass of Arm26 model (Figure 7A). With the increase of lower arm mass by 1 kg (second scenario, Figure 7B), all the targets could still be reached, although it presents more oscillatory trajectories that go further down reflecting the weight increment under gravity effect. In comparison, for the scenarios with the simpler spinal cord network that does not consider spindle proprioception, not all targets could be reached, even though less weights had to be optimized compared to the complete spinal cord network. In the third scenario without perturbation (Figure 7C), four of the eight targets were successfully reached [*T*5 (green), *T*6 (light blue), *T*7 (red) and *T*8 (pink)], with the target *T*3 (yellow) being also almost reached. The scenario with perturbation (Figure 7D) presents a performance reduction compared to the previous scenario, not reaching the target *T*5 (green) anymore—although close—, and with a more distant trajectory from the target *T*3 (yellow). The reached targets in these latter scenarios using a simpler spinal network are the lower targets, which benefit from gravity and, therefore, required less muscle effort in comparison to higher above placed targets.

Figure 7 also allows to observe the timing characteristics of the reaching hand-trajectories, i.e. how the trajectories behaved over time. The timing characteristics of the movement are illustrated by overlaid black dots on reaching trajectories which are spaced 50 ms apart, thus, separating the 0.8s-simulated trajectories in 16 50 ms-gaps. Therefore, it is possible to inspect the movement velocity, based on the distance between dots: close dots represent slower movement (long time in a short trajectory space), while distant dots mean faster movement (less time to travel a greater distance). In the first scenario (Figure 7A), the points are close in the beginning of trajectories, more distant in the middle, and close again at the end as approaching the targets. This corresponds to a bell-shaped velocity characteristic along the path as observed during human reaching movement from one point to another (Morasso, 1981). Most of trajectories in the other scenarios present a similar velocity behavior, however, some trajectories in the scenarios with simpler spinal cord circuitry exhibit a greater distance between the last dot and the target [*T*3 (yellow) in Figure 7C, and *T*6 (light blue) in Figure 7D], implying a higher velocity at the end of movement.

Additionally, in the Supplementary Figures S1–S8, it is possible to further observe the resulting movements, with the hand-trajectories split into *X* and *Z* coordinates over the entire simulation time, considering the four scenarios for each of the eight targets. The trajectories before completion of optimization (first iteration of the learning algorithm) and after completion of optimization (best solution found by the algorithm) are displayed. The after trajectories correspond to the trajectories presented here in the Figure 7. The reaching performance is also quantitatively demonstrated by including the cost-function (*J*) values from Equations 2, 3, both previous and posterior the optimization end. Furthermore, the Supplementary Figure S9 shows the learning curves, i.e. how the best cost value develops over the optimization iterations of the CMA-ES algorithm, for the four evaluated scenarios. We present the learning curves for the target *T*1 (which was reached in the scenarios with complete spinal circuitry model, and not in the ones with simpler spinal circuitry model) and target *T*3 (which was reached in the scenarios with complete spinal circuitry model, and almost reached in the scenario with simpler spinal circuitry model without perturbation, but not with perturbation).

#### 3.2.2 Spinal synaptic weights

Regarding the analysis of the optimized synaptic weights in the spinal neuronal connections, this was performed observing the weight values after optimization. We created connection matrices with the spinal synapses for each of the eight evaluated targets in the four different scenarios, in order to observe by a color-map whether the synapses became stronger or weaker, based on weight values, as result of the optimization process for the center-out reaching sensorimotor task.

The connection matrices were created displaying the presynaptic and postsynaptic units from the spinal cord circuitry, respectively in the vertical and horizontal axes. The presynaptic units in the complete spinal cord model correspond to alpha, dynamic gamma and static gamma motoneurons, together with Ia, propriospinal and Renshaw interneurons, including Ia and II afferents from the muscle spindle model that innervate spinal neurons (see Section 2.2). These presynaptic units were placed in the matrices following the just mentioned order, presenting each neuronal type at a time for all the six MTUs (from muscle 1 to muscle 6: MEF, MEE, BEFSA, BEESR, MSA and MSR), totalizing 48 units. The postsynaptic units correspond to the same as presynaptic units, excluding the Ia and II afferents. They were displayed in the same order as previously, also for all the six MTUs at a time, totalizing 36 units. The simpler spinal cord model that does not include spindle proprioception has only alpha motoneurons and Renshaw interneurons as presynaptic and postsynaptic units. They were disposed in the connection matrices following the previous order, again for all the six MTUs at a time, totalizing 12 presynaptic and postsynaptic units.

**Figure 8:**
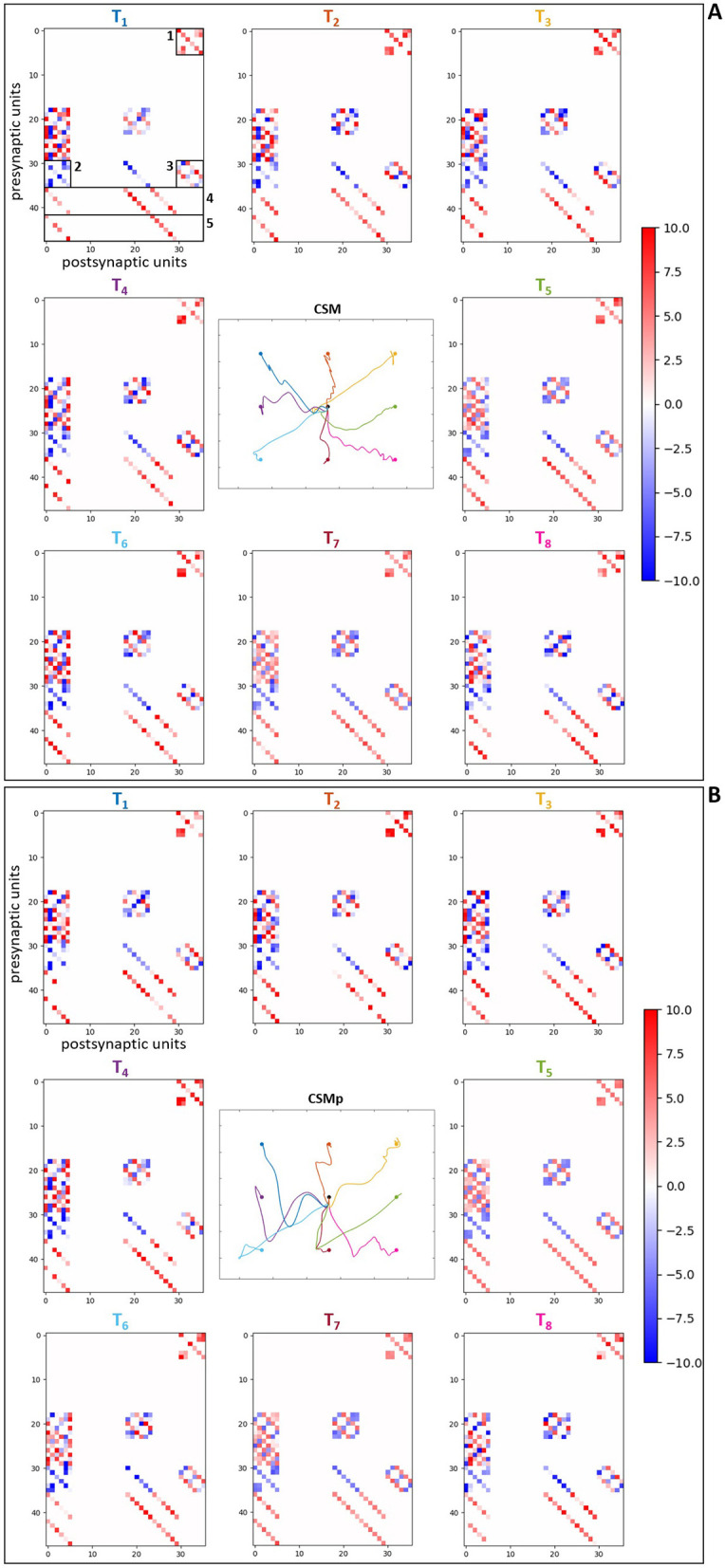
Matrices of final synaptic weights for complete spinal circuitry model. **(A)** Without perturbation. **(B)** With perturbation. The resulting weights from the individual optimizations for the eight evaluated targets are presented as color-map matrices (placed according to the targets' position), together with a reduced version of the resulting trajectories from Figure 7 showing whether each set of weights depicted led to a successful task. In all the matrices, the presynaptic units are displayed in the vertical axis for the six MTUs, in the order: alpha, dynamic gamma and static gamma motoneurons, Ia, propriospinal and Renshaw interneurons, followed by Ia and II spindle afferents, totalizing 48 units. The postsynaptic units are displayed in the horizontal axis for the six MTUs, in the same order as before, excluding the Ia and II spindle afferents, totalizing 36 units. 1, 2 and 3 in **(A)** correspond to the connections also present in the simpler spinal cord model: 1—synapses from alpha motoneurons to Renshaw interneurons, 2—synapses from Renshaw interneurons to alpha motoneurons, 3—synapses between Renshaw interneurons. 4 and 5 correspond to the Ia and II afferent connections, respectively.

**Figure 9:**
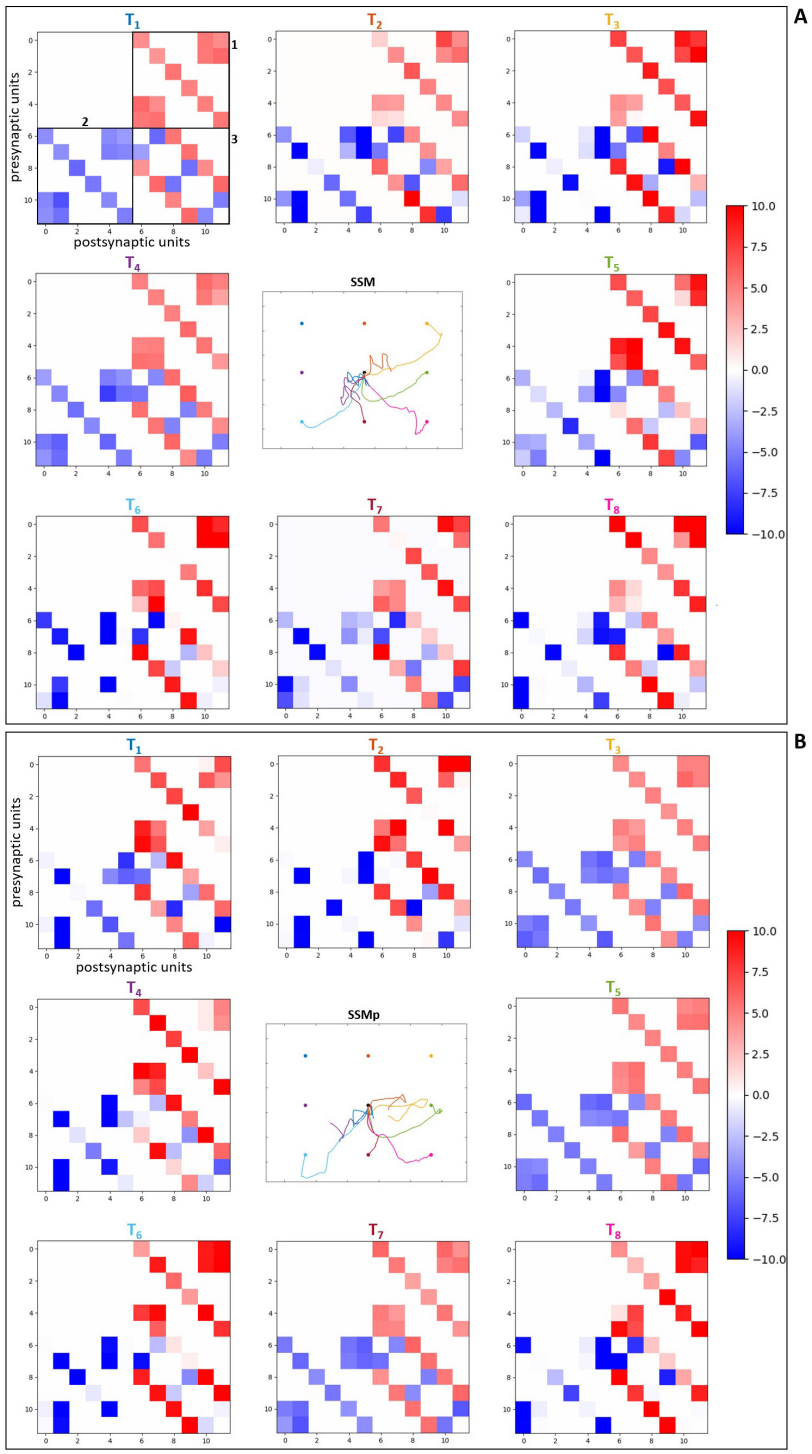
Matrices of final synaptic weights for simpler spinal circuitry model. **(A)** Without perturbation. **(B)** With perturbation. The resulting weights from the individual optimizations for the eight evaluated targets are presented as color-map matrices (placed according to the targets' position), together with a reduced version of the resulting trajectories from Figure 7 showing whether each set of weights depicted led to a successful task. In all the matrices, the presynaptic units are displayed in the vertical axis for the six MTUs, from alpha motoneurons to Renshaw interneurons, totalizing 12 units. The postsynaptic units are displayed in the horizontal axis for the six MTUs, corresponding to the same 12 units as presynaptic ones, also placed in the same order. 1, 2 and 3 in **(A)** correspond to the connections highlighted in Figure 8A: 1—synapses from alpha motoneurons to Renshaw interneurons, 2—synapses from Renshaw interneurons to alpha motoneurons, 3—synapses between Renshaw interneurons.

The color-map in the matrices allows to differentiate the synaptic weights as excitatory (positive, red) and inhibitory (negative, blue), as well as how high/low their final values are by the color intensity, i.e. it is possible to observe how strong the synapses are after optimization. Therefore, when we compare the matrices for the scenarios with complete spinal cord model without and with perturbation (Figures 8A, B, respectively), we notice that the spindle connections (indicated by 4 and 5 in Figure 8A) were intensified for the most difficult targets against gravity in the scenario with perturbation [*T*1 ([0.23, –0.23]m), *T*2 ([0.33, –0.23]m), *T*3 ([0.43, –0.23]m) and *T*4 ([0.23, –0.33]m) in Figure 8B]. These targets correspond to the higher ones which the simpler spinal cord model (without spindle afferents) was not able to reach after optimization. Thus, the spindle afferents' synapses, with increased weight values, seem to play an important role to reach these difficult targets under perturbation. It is observed that the connections present in the scenarios with simpler spinal cord circuitry were also intensified for these difficult targets [except for the target *T*3 ([0.43, –0.23]m)] when comparing without and with perturbation (Figures 9A, B, respectively). However, as shown in the previous section, even with stronger synapses, the limited number of connections (also not including spindle proprioception) was not sufficient to make the simpler spinal circuitry succeed in reaching such targets.

Furthermore, for some targets the weight values were suppressed (closer to zero, represented by lighter colors) in both complete (Figure 8) and simpler (Figure 9) spinal cord models when comparing them between the scenarios without and with perturbation. These targets are *T*5 ([0.43, –0.33]m) and *T*7 ([0.33, –0.43]m), which seem to be facilitated to reach by the increase of lower arm mass, demonstrated by the synapses that do not need to be strong for the reaching task. The above observations are supported by the Supplementary Figures S10, S11, which complement the Figure 8, Figure 9, respectively, by presenting histograms with the final values of synaptic weights.

Additionally, to reinforce the observation from the analyses of weight matrices, that the spindle connections (indicated by 4 and 5 in Figure 8A) were intensified for the difficult higher targets [*T*1 ([0.23, –0.23]m), *T*2 ([0.33, –0.23]m), *T*3 ([0.43, –0.23]m) and *T*4 ([0.23, –0.33]m)] when comparing the scenarios without and with perturbation, we constructed histograms only with the final values of synaptic weights from spindle afferent connections for these difficult targets in the scenarios with complete spinal cord model, both without and with perturbation (Figures 8A, B, respectively). Therefore, we can notice a stronger agglomeration of weights at the top values for excitatory synapses (between 7 and 10) when the perturbation is included and compared without it. This shows that the spindle afferent synapses were strengthened for these difficult targets under perturbation, highlighting the importance of the spindle afferents to succeed during the task.

## 4 Discussion

### 4.1 Spinal cord model

The scientific community has recognized the importance of combining knowledge of different disciplines—from behavioral studies, neurophysiological investigations and computational modeling—to understand the processes occurring within the CNS and the role of local circuits in the spinal cord during the execution of voluntary movement (Alstermark et al., 2007). Computational modeling allows the investigation of neural pathways that could not be explored in neurophysiological studies, e.g., the addition of neuronal connections or exclusion of local circuits as performed in this study. In this context, over the past 15 years, various authors have adopted computational models of the spinal cord to reproduce different motor tasks (Raphael et al., 2010; Tsianos et al., 2014; Buhrmann and Di Paolo, 2014; Elias et al., 2014; Stefanovic and Galiana, 2015; Li et al., 2015; Teka et al., 2017; Parziale et al., 2020; Verduzco-Flores and De Schutter, 2022; Bruel et al., 2024a). They have observed that the mammalian spinal cord appears to provide a high-dimensional control space that facilitates the rapid and successful learning of new motor tasks. Therefore, studies like the one presented here, which investigate spinal cord circuitry, are relevant for a better comprehension of sensorimotor control.

According to (Raphael et al. 2010) and (Tsianos et al. 2014), the way they established the connections in the network, following known spinal pathways (Pierrot-Deseilligny and Burke, 2005), contributed to the successful performance in their studies. As highlighted by (DeWolf et al. 2016)—who did not include a spinal cord model in their spiking neural model for arm control—, the inclusion of dynamics in the musculoskeletal system together with details of spinal cord circuitry would improve the overall biological realism of their model. Here we chose to implement physiological connections in the spinal cord model in order to maintain a biological level of detail sufficient to observe the processing of spindle proprioception by spinal circuitry. Therefore, we implemented classic types of interneurons together with alpha motoneurons—representing neural interactions between the simulated muscles—, including dynamic and static gamma motoneurons that were not modeled in the previous studies.

As concluded in the modern view about spindle roles by (Dimitriou and Edin 2010), muscle spindles can act as *forward sensory models* (Miall and Wolpert, 1996), i.e. they are influenced by the current state of their parent muscle, as well as by the fusimotor control from gamma motoneurons, with their discharges representing future kinematic states. This finding suggests a novel function of gamma motoneurons, which should be considered in theories of motor learning. If this view is correct, the fusimotor drive would be involved in the orchestration of complex motor actions across multiple muscles and joints. Therefore, the CNS is expected to learn how to control both the skeletal muscles, by alpha motoneurons' activation, and the fusimotor system, by gamma moroneurons' activation. This highlights the importance of including gamma moroneurons in modeling of sensorimotor learning as novelly implemented here.

Furthermore, as observed by (Verduzco-Flores and De Schutter 2022), most of the current models for reaching and motor control do not use neural systems to perform motor commands and they do not model a biological synaptic learning. Physiologically-inspired spiking neural networks require neuronal models that are able to replicate both observable behavior and spiking activity comparable to experimental data (DeWolf et al., 2016). Typically, works on the motor system focus either on behavioral aspects or on neural aspects. The current work combines both aspects, utilizing spiking neurons in NEST to construct physiological spinal circuitry and performing behavioral reaches using musculoskeletal model in demoa.

However, our modeling approach of the spinal circuitry also has limitations arising from simplifying assumptions. First, the spiking neural network simulator, NEST, corresponds to a point-neuron (i.e. single compartment neuron) simulator; therefore, it represents the neurons without axons and dendrites, not allowing to model morphological and biophysical details of individual neurons (Gewaltig and Diesmann, 2007). The simulator is rather intended to model dynamics and structure of neural networks, as the physiological spinal pathways reproduced here. Although, it allowed us to explore learning and plasticity in a model of sensorimotor processing, the simulator does not enable to conduct further investigations related to morphology of the neurons in spinal cord. For example, a significant effect on muscle force generation was demonstrated from persistent inward currents into dendritic compartments of motoneuronal models, modifying the motor control for a desired condition (Elias et al., 2012; Elias and Kohn, 2013).

Second, the spinal circuitry developed in this work has a limited number of neurons. Each neuronal unit in our model represents the activity of the neuronal population responsible for each modeled muscle. This may neglect fine-grained computations from spike timing of several neurons. As well, the proportion for different neuronal types is not taken in consideration. Third, we chose to not include the group II interneurons receiving the spindle II afferent and exciting the homonymous alpha motoneurons—due to the lack of this neuronal type in most of the works reviewed and for modeling simplification—, implementing only the monosynaptic connection between II afferents and alpha motoneurons in our complete spinal cord circuitry. However, this pathway could also be explored as implemented in (Elias et al. 2014) for postural control task, representing an additional learning circuit in the spinal cord. Furthermore, if a model of GTO was included (e.g., Mileusnic and Loeb, 2006), as this sensor has been indicated to improve joint-based control (Kistemaker et al., 2013), it would enable to expand the spinal circuitry by the addition of the inhibitory Ib pathway. Nevertheless, it has been recently observed that such pathway did not lead to robustness against perturbation (Bruel et al., 2024b), as differently highlighted in previous work (Kistemaker et al., 2013).

On the other hand, when modeling the CNS, most of the time we do not know in advance the appropriate level of detail that should be considered. In this context, exploring plausible levels related to intended behavior could most efficiently lead to a good understanding of the structure-to-function relation (Eliasmith and Trujillo, 2014). The fundamental purpose of neuroscience is to comprehend the relation between brain and behavior. Therefore, while modeling, a clear link to behavioral constraints must be established, in a way that the model details should depend on the targeted questions. Here, the modeling details included fulfill our research objective of connecting the muscle spindle model to a physiologically-motivated spinal circuitry, aiming to investigate if the processing of afferent firings by the spinal network influences performance during sensorimotor task.

Finally, the absence of higher-level CNS models—although in accordance with our motivation of forcing the spinal circuitry to generate all the required dynamics to succeed in reaching task—, represents a simplification of the sensorimotor system that includes other structures not considered in this study. These structures largely interplay to achieve the ultimately complex motor behavior. For reaching movement, it has been previously demonstrated that the spinal cord is sufficient to deal with the task complexity (Raphael et al., 2010; Tsianos et al., 2014). However, for more complex tasks, further CNS parts should be included in the neuronal modeling. For instance, it has been attributed to cerebellum the responsibility of developing and storing internal models of the system dynamics, and generating adaptive signals for error correction (Schweighofer et al., 1998; Kawato et al., 2003). Moreover, it has been shown that the primary motor cortex has cells sensitive to the activation of specific muscles, and cells sensitive to the general direction of movement (Griffin et al., 2015).

### 4.2 Sensorimotor task

As reported by (Parziale et al. 2020), it remains an open question how the brain controls voluntary arm movements to reach a point (i.e. target) in space or to produce a complex movement. In the last decades, significant effort has been devoted to elucidate the neural mechanisms involved in this process (Georgopoulos et al., 1982; Bonfiglioli et al., 1998; Maynard et al., 1999; Smith and Shadmehr, 2005; Ashe, 2005; Bizzi and Ajemian, 2015). In this context, studies like the one conducted here, which use computational modeling to investigate the neural mechanisms involved in motor control, are important because computational models can overcome the technical difficulties encountered during real-world tasks. For example, behavioral studies in humans need to be conducted under controlled conditions, with the individual performing a limited set of movements. Additionally, there are studies considered impractical or immoral to be executed on living subjects over the long-term (van Ooyen, 2011).

#### 4.2.1 Reaching trajectories

In this work, a 2D musculoskeletal model was used. This approach might be considered limited since natural human reaching movements occur in 3D space, aligned to more complex muscle arrangements and postural complications. However, 2D control approaches are still relevant because all human hand movements are executed within the elbow plane, with a variable reference frame based on shoulder rotation (Stefanovic and Galiana, 2014). Furthermore, due to the complications related to posture and gravity when studying 3D movements, most of the previous studies that investigated human reaching movement by neural modeling used 2D models. Additionally, regarding the adopted reaching task—starting from a central position to peripheral targets in different directions (center-out reaching task)—, it is a standard clinical behavioral test that has been largely used in experimental studies as well (e.g., Georgopoulos et al., 1982; Fu et al., 1993; Ashe and Georgopoulos, 1994), allowing further comparison.

As defined by (Morasso 1981) and presented in further studies (Flash, 1987; Flanagan et al., 1993; Churchland et al., 2006), the human reaching movement typically follows a straight path with a bell-shaped velocity profile. However, after optimization, the simulated trajectories in the current work showed oscillations (see Figure 7). Although the literature often describes movement trajectories as straight lines, actually there is noticeable curvature in experimentally observed reaching trajectories across various contexts (Morasso and Ivaldi, 1982; Morasso, 1983; Flash and Hogan, 1985; Graham et al., 2003; Charles and Hogan, 2010). Specifically, (Graham et al. 2003) reported that movement profiles exhibit significant curvatures that are distinct in each direction. (Flash and Hogan 1985) also demonstrated through empirical data the presence of uni- or bimodal curves and hooks at the end of the movement. Moreover, other studies investigating reaching movements using spinal cord modeling have also shown curved trajectories (Stefanovic and Galiana, 2014; Buhrmann and Di Paolo, 2014; Verduzco-Flores and De Schutter, 2022).

(Verduzco-Flores and De Schutter 2022) reported that their reaching movements are ataxic (with lack of coordination), according to a motor system without a cerebellum. They compared their model to the brain of a baby who performs clumsy reaching movements, as at birth the cerebellum is not fully-developed and probably not functional. Similarly, without a cerebellum model, our reaching trajectories are ataxic movements, presenting oscillations and undershooting or overshooting the target before reach (Becker and Person, 2019). (Day 1998) examined reaching movements in cerebellar patients. They observed longer, more circuitous trajectories—similar to our simulation outcome—, with most changes in direction during the last quarter. Patients with cerebellar ataxia are not able to adjust to tasks that require a fast reaction; however, as demonstrated by (Richter et al. 2004), they are still able to learn how to move in a novel dynamic environment, where there is a remapping of afferent inputs to movement as performed in this study.

#### 4.2.2 Learning algorithm

In order to perform the remapping of afferent inputs, i.e. learning and plasticity in sensorimotor processing, we adopted the CMA-ES algorithm, which corresponds to an evolutionary computational method (Hansen, 2016). As stated by (Loeb 2012), biological systems use trial-and-error learning in order to obtain a collection of useful sensorimotor behaviors. Applying the evolutionary algorithm, we implemented this trial-and-error process by optimizing the spinal connections, thereby regulating the activity of the spinal cord circuitry. Evolutionary algorithms have been previously used for parameter calibration of neural computational models (Antonietti et al., 2016; Buhrmann and Di Paolo, 2014). Specifically, (Parziale et al. 2020)—who also implemented known spinal pathways from literature to investigate reaching movements—adopted a type of evolutionary algorithm (Storn and Price, 1997) to perform the trial-and-error process for regulation of their spinal cord network. They observed that the algorithm is fairly fast and efficient in finding the global solution, aligned to few control parameters. Similarly, in the present work, we found efficient solutions to the high-dimensional optimization problem (see Section 3.2.1) performing a relatively fast computation (maximum of 1,000 iterations).

Therefore, the CMA-ES algorithm represents a good alternative for the gradient descent algorithm adopted in the studies that motivated the spinal cord circuitry of this work (Raphael et al., 2010; Tsianos et al., 2014). Although their learning algorithm was also capable of finding good solutions for the task, local optima can hinder the effectiveness of gradient-based algorithms (Buhrmann and Di Paolo, 2014). Additionally, as highlighted by (Ruder 2016), because gradient algorithms are sequential (advancing step by step toward the minimum), they provide good convergence but might be slow, especially with large datasets. This is particularly critical to the scenario presented here, where a large number of synaptic weights were optimized.

We conducted new optimization for each individual target in the four evaluated scenarios (see Section 2.3.1). (Loeb 2012) concluded that every time that a similar task has to be executed by biological systems, the previously learned collection of motor commands is recalled, facilitating the task achievement. Thus, to execute a new task, we could use a close previously-learned task and slightly adjust for the novel goal (Loeb, 1983). Consequently, to reach a new close target, most probably the CNS rather perform minor refinements (e.g., recruiting different muscle fibers), instead of learning from scratch the necessary motor skills to succeed in the task. In our modeling, there is only one muscle fiber aligned to a single spindle per muscle; therefore, it is not possible to recruit different fibers and spindles inside muscles in order to change movement. In this scenario with simplified details of the musculoskeletal model, it seems sensible that the neural model has to compensate, learning the synaptic weights to reach every new target.

#### 4.2.3 Synaptic weights

**Figure 10:**
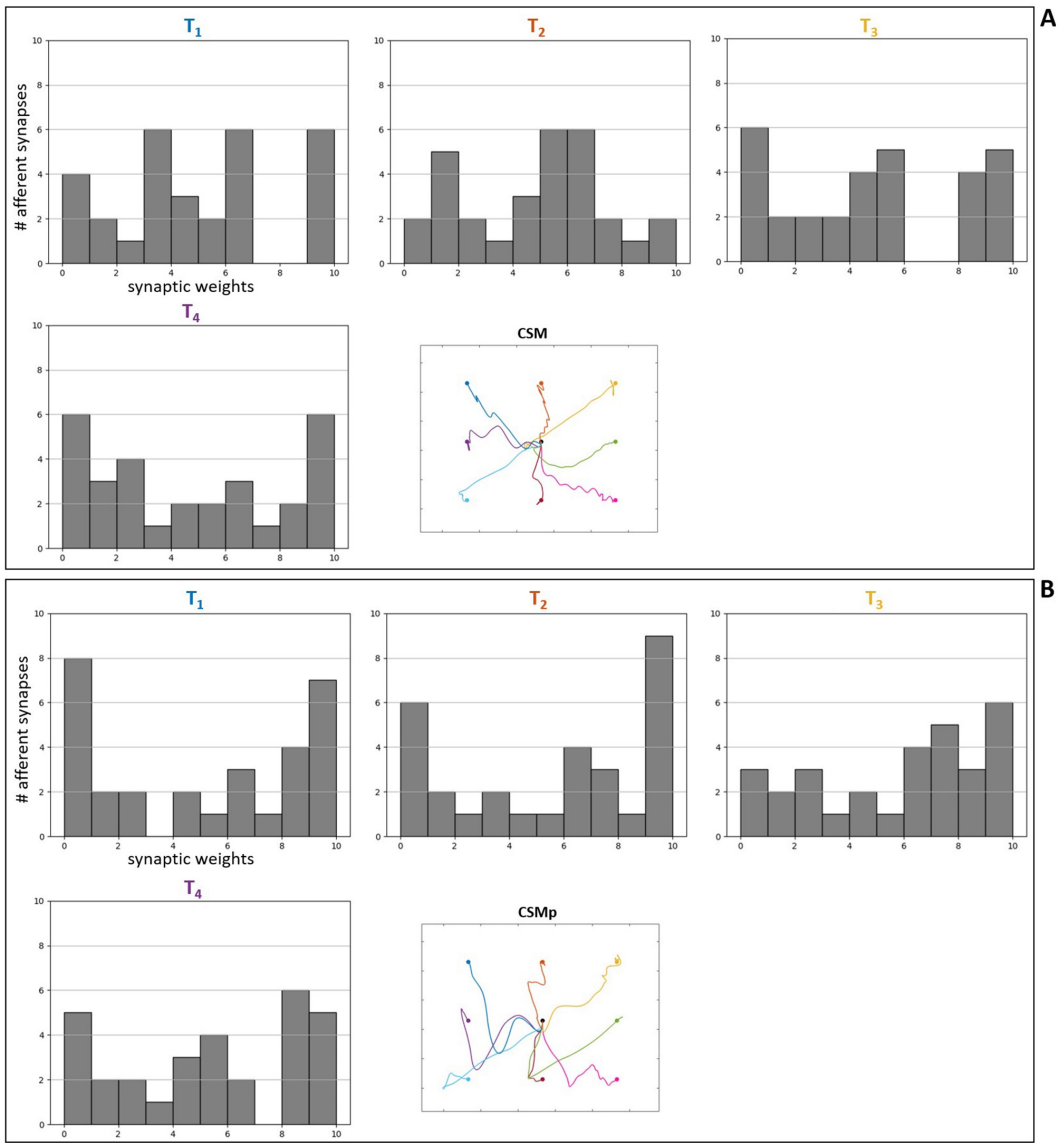
Histograms of final synaptic weights for afferent spindle connections in the complete spinal circuitry model. **(A)** Without perturbation. **(B)** With perturbation. The final weight values of spindle synapses from the individual optimizations for four of the evaluated targets [*T*1–*T*4— most difficult (higher) targets under gravity] are presented as histograms, according to the targets' position, together with a reduced version of the resulting trajectories from Figure 7 for reference.

Previous experiments showed the presence of plasticity mechanisms dependent on activity in the spinal cord of rats, primates and humans (Wolpaw, 2010). Moreover, spinal synaptic strength can vary between pathways and movement tasks (e.g., Yavuz et al., 2018). We focused the analyses of spinal connections on synaptic weight modification/strength after optimization, similar to what was performed in other recent studies (Bruel et al., 2024a,b) to evaluate the role of synaptic connections in motor learning. Motivated by proprioception investigation, we specially analyzed the spindle afferent connections in the spinal network. We found that these connections were intensified for the higher targets (considered more difficult under gravity) when comparing the scenarios without and with perturbation (increment of lower arm segment in 1 kg) (Figures 8, 10). Therefore, the muscle spindle connections were strengthened for difficult targets under perturbation, highlighting the importance of spindle proprioception for the task success.

This finding is in line with previous works that demonstrated the relevance of spindle proprioception for movement performance. It has long been argued that spindle afferent feedback benefits the dynamic stiffness and damping of muscle, helping to stabilize posture and movement (Nichols and Houk, 1976; Hogan, 1985). (Rodrigues and Elias 2019) showed by computer simulations that anatomical (quantity) and functional (synaptic conductance) loss of Ia afferents—as observed in peripheral neuropathies— significantly decreases short-latency stretch reflex. In addition, proprioceptive and visual information are indicated to be weighted according to their direction-dependent precision during reaching movements (van Beers et al., 1999). As pointed out by (Dimitriou 2022), spindles and their fusimotor control may facilitate internal coordinate transformations to succeed in a new task. He highlighted the importance of further works examining spindle afferent during dynamic (i.e. perturbed) learning, because he has previously observed substantial modulation of spindle afferent signals as a function of adaptation state during center-out reaching experimental task (Dimitriou, 2016). More recently, a muscle spindle signal based controller, implemented by (Stollenmaier et al. 2020), contributed to the muscle stimulations during two DoFs point-to-point arm movements mostly in the presence of perturbation (external forces).

Alternatively to the optimization of spinal synaptic weights, the supraspinal commands (descending commands) could be optimized, which here corresponded to a constant input current set to the spinal neurons (see Section 2.2). (Bruel et al. 2024b) optimized on one hand the supraspinal commands, and on the other hand the spinal synaptic strengths, using the same CMA-ES algorithm adopted here. They concluded that both strategies could reproduce similar trajectories in different gravity environments. Such strategy of modulation of the supraspinal commands could make up for the simpler spinal cord circuitry (including only Renshaw pathway, without spindle proprioception) that did not succeed in the task of reaching all the evaluated targets (Figure 7). In a scenario with limited low-level sensorimotor control, it seems plausible that the higher commands would have to compensate for movement performance. (Gizzi et al. 2021b) suggested that a compensation of the higher center's drive may be necessary to maintain a motor task during blood flow restriction that led to decrease in the Ia and II afferent activity.

Additionally, in their “minimal” spinal cord model, (Bruel et al. 2024b) chose to include only the stretch reflex and inhibitory Ia pathways—also implemented in the complete spinal circuitry of the present study—as these pathways (both conveyed by Ia spindle fibers) demonstrated the more notable effect on motor control in terms of kinematics and muscle recruitment. Therefore, it is not clear if a spinal cord circuitry that only includes Renshaw pathway would succeed in reaching all targets with modulation of the supraspinal commands. At this point, it is relevant to highlight that we modeled a complete Renshaw pathway, including the Renshaw cell connections to antagonist Ia interneurons as well as antagonist Renshaw cells, which were absent in the previous work (Bruel et al., 2024b) and specifically mentioned as spinal connections that could be further explored in their model.

## 5 Conclusion

In this study, we developed a combined model of spinal cord physiology and musculoskeletal biomechanics, with a particular focus on integrating classic spinal interneurons and both alpha and gamma motoneurons. This biologically detailed approach allowed us to examine how spinal circuits process proprioceptive inputs from muscle spindles and highlighted the potential contribution of gamma motoneurons to anticipatory motor control and learning. The findings emphasize the value of including gamma motoneurons in computational models of sensorimotor learning.

By constructing the spinal circuitry using spiking neurons and coupling it to a musculoskeletal arm model, we were able to simulate goal-directed reaching movements and probe the neural mechanisms underlying motor control. The use of an evolutionary learning algorithm (CMA-ES) enabled us to mimic trial-and-error motor learning, revealing solutions that adapt neural connectivity for the successful execution of reaching tasks, especially under perturbed conditions such as increased load.

Nonetheless, our approach includes several limitations. The use of point-neuron models precludes the detailed representation of dendritic and axonal morphology, which are known to influence motoneuron firing and muscle force generation. Additionally, the reduced number of neuronal units and lack of heterogeneity among neuronal types limit the granularity of the simulation. Simplifying assumptions, such as omitting group II interneurons and not modeling higher-order CNS structures, restrict our ability to generalize the findings to more complex motor behaviors.

Despite these limitations, our results demonstrate the critical role of muscle spindle connectivity and proprioceptive integration in motor performance. The strengthening of spindle afferent connections for more demanding tasks under load underscores the adaptive capabilities of spinal circuitry in sensorimotor control. Computational modeling, as showcased here, remains an essential tool for uncovering the neural underpinnings of movement, particularly when direct experimental exploration is technically challenging.

Future work should address current limitations by incorporating more detailed neuron models, expanding the size and diversity of spinal circuits, and embedding higher-level neural structures to simulate more complex motor behaviors. These directions will further bridge the gap between computational models and the rich dynamics of biological motor control.

## Data Availability

The datasets presented in this study can be found in online repositories. The names of the repository/repositories and accession number(s) can be found in the article/Supplementary material.

## Footnotes

^1^https://doi.org/10.18419/DARUS-2550.

^2^https://nest-simulator.readthedocs.io.
