# Supplementary material for: Closed-loop coupling of both physiological spindle model and spinal pathways for sensorimotor control of human center-out reaching
Source: Front Comput Neurosci. 2025 Aug 26;19:1575630. doi: 10.3389/fncom.2025.1575630 (PMC12417497; doi:10.3389/fncom.2025.1575630)
Supplement: Supplementary file 1 [file Data_Sheet_1.pdf]

Supplementary Material - Closed-loop coupling of both physiological spindle model and spinal pathways for sensorimotor control of human center-out reaching

Pablo Filipe Santana Chacon [1], Isabell Wochner [2,3], Maria Hammer [1], Jochen Martin Eppler [4], Susanne Kunkel [4,5] and Syn Schmitt [1,3,6,*]

[1]Institute for Modelling and Simulation of Biomechanical Systems, University of Stuttgart, Stuttgart, Germany
[2] Hertie Institute for Clinical Brain Research, University of Tübingen, Tübingen, Germany
[3]Center for Bionic Intelligence Tübingen-Stuttgart (BITS), Tübingen-Stuttgart, Germany
[4]Faculty of Science and Technology, Norwegian University of Life Sciences, Ås, Norway
[5]Peter Grünberg Institute (PGI-15), Jülich Research Centre, Jülich, Germany
[6]Stuttgart Center for Simulation Science, University of Stuttgart, Stuttgart, Germany

*Syn Schmitt - schmitt@imsb.uni-stuttgart.de

1 SUPPLEMENTARY DATA

The implemented version of the Arm26 model in demoa, together with instructions of how to run with python, muscle spindle module and NEST simulator, as well as the scripts for coupling both simulators and optimizing the spinal synaptic weights during center-out reaching task, have been made available at `https://doi.org/10.18419/DARUS-4765`.

2 SUPPLEMENTARY FIGURES

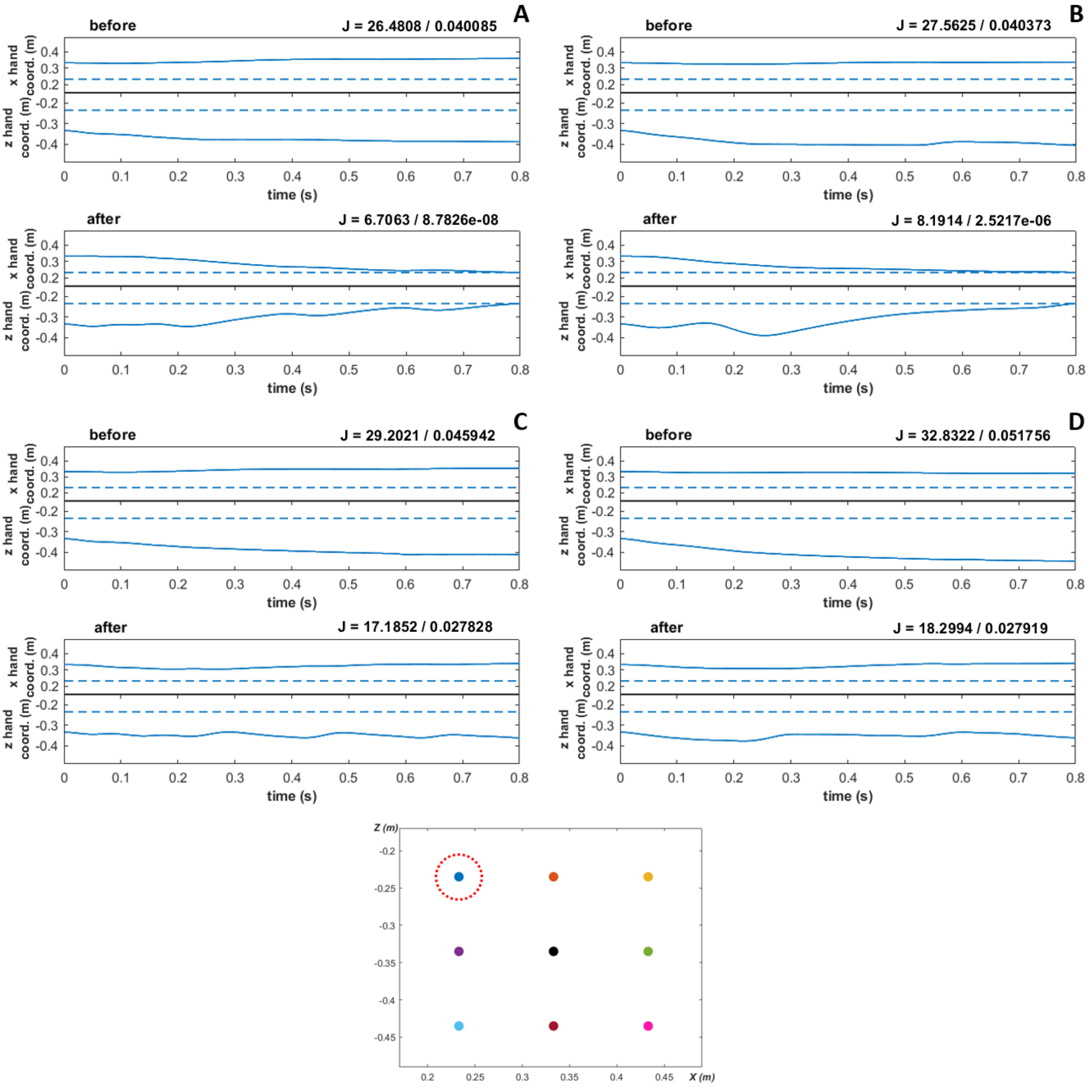

Figure S1. Reaching hand-trajectory per coordinate versus time – target $T1$ ([0.23,-0.23]m). Before completion of optimization (first optimization iteration) and after completion of optimization (best solution found). J corresponds to the value of cost functions used during optimization, with the first value representing the sum of Euclidean distances between all points of the simulated trajectory to the target, and the second value showing the Euclidean distance only between the final point of simulated trajectory and the target. Continuous lines correspond to the hand trajectory, and dashed lines correspond to the target coordinate. **A**: Complete spinal circuitry model without perturbation. **B**: Complete spinal circuitry model with perturbation (increment of lower arm segment in 1 kg). **C**: Simpler spinal circuitry model (without spindle proprioception – only Renshaw pathway) without perturbation. **D**: Simpler spinal circuitry model with perturbation. For reference, the evaluated target is indicated in the bottom.

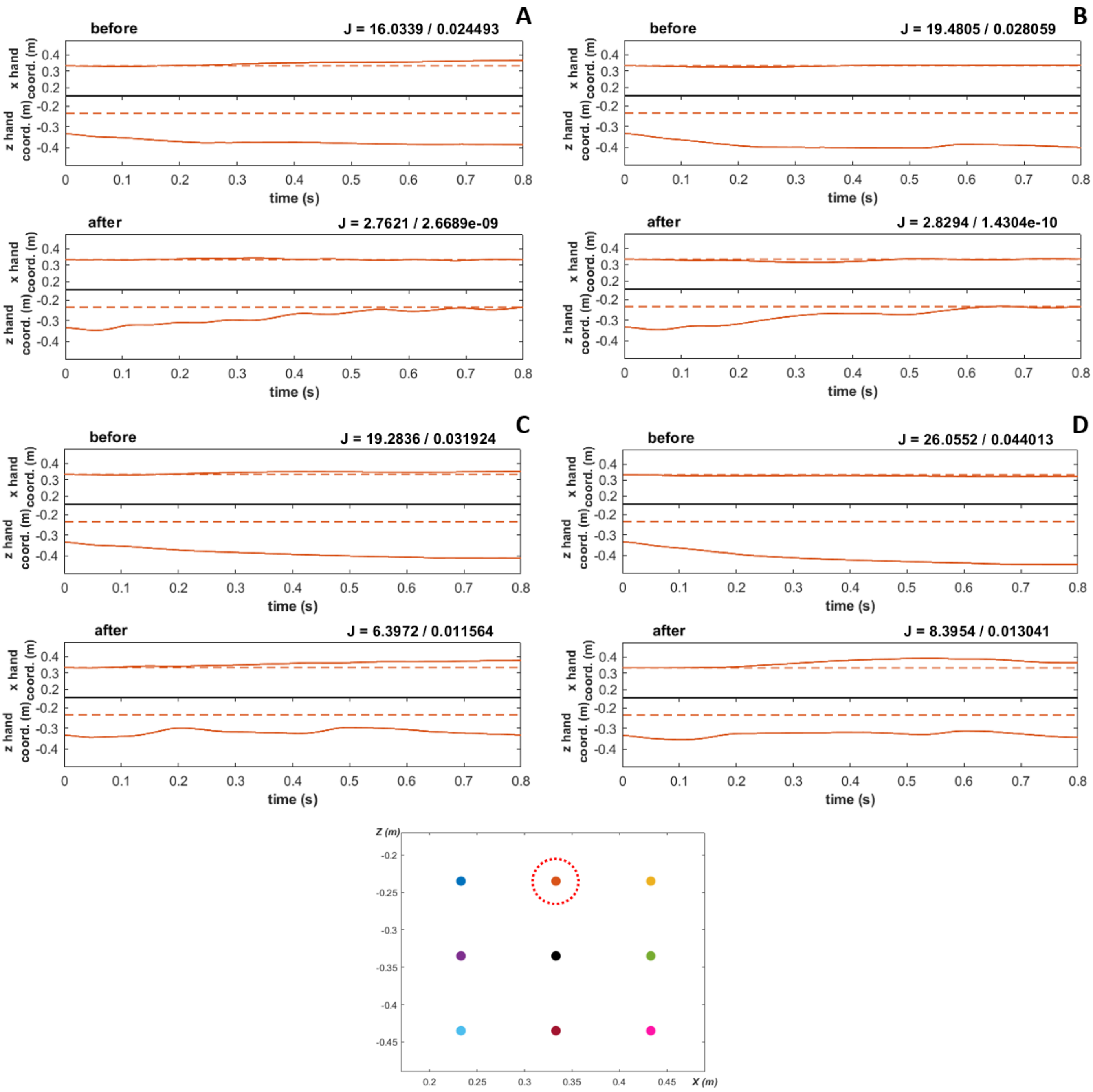

Figure S2. Reaching hand-trajectory per coordinate versus time – target $T2$ ([0.33,-0.23]m). Before completion of optimization (first optimization iteration) and after completion of optimization (best solution found). J corresponds to the value of cost functions used during optimization, with the first value representing the sum of Euclidean distances between all points of the simulated trajectory to the target, and the second value showing the Euclidean distance only between the final point of simulated trajectory and the target. Continuous lines correspond to the hand trajectory, and dashed lines correspond to the target coordinate. **A**: Complete spinal circuitry model without perturbation. **B**: Complete spinal circuitry model with perturbation (increment of lower arm segment in 1 kg). **C**: Simpler spinal circuitry model (without spindle proprioception – only Renshaw pathway) without perturbation. **D**: Simpler spinal circuitry model with perturbation. For reference, the evaluated target is indicated in the bottom.

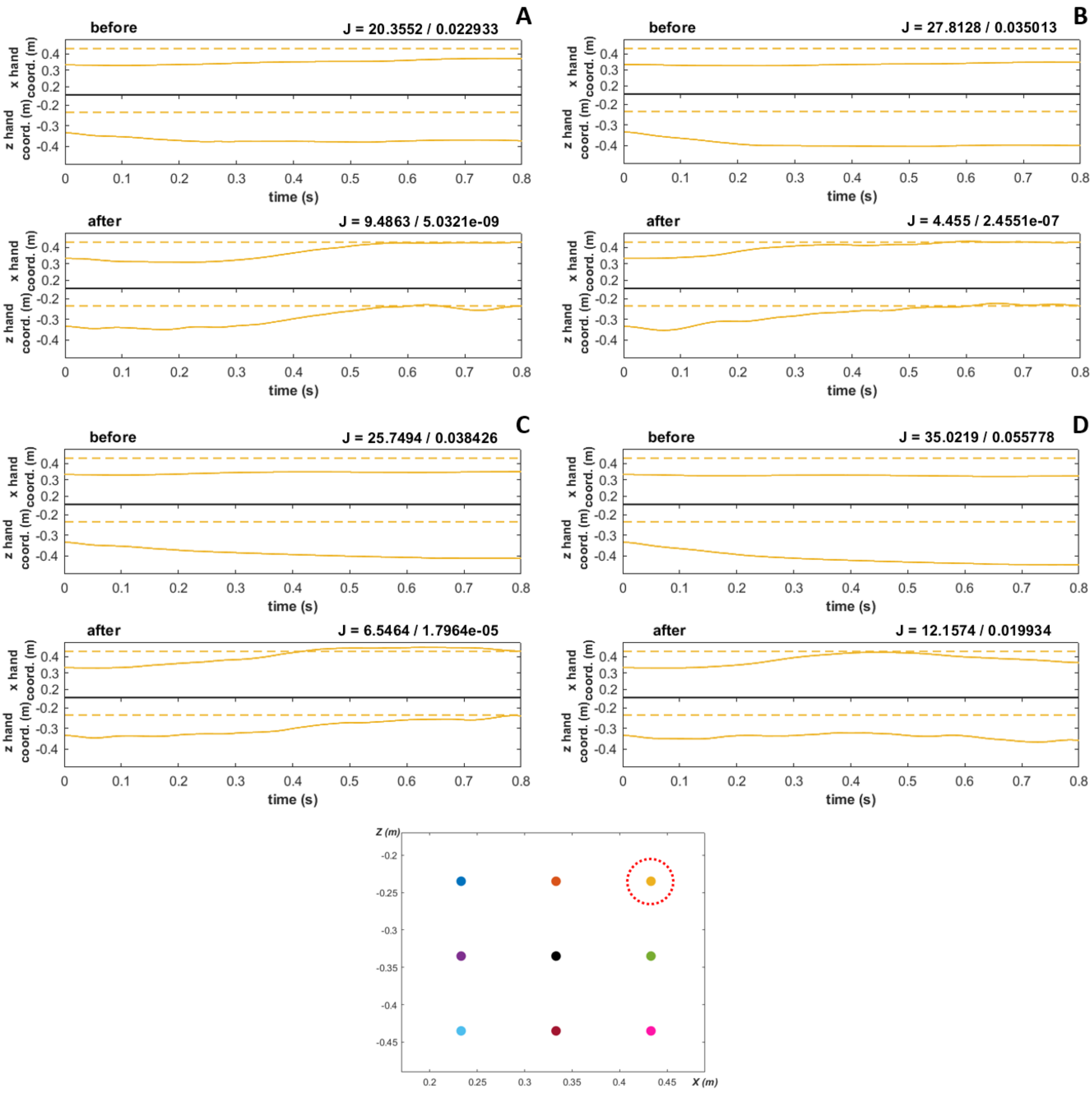

Figure S3. Reaching hand-trajectory per coordinate versus time – target $T3$ ([0.43,-0.23]m). Before completion of optimization (first optimization iteration) and after completion of optimization (best solution found). J corresponds to the value of cost functions used during optimization, with the first value representing the sum of Euclidean distances between all points of the simulated trajectory to the target, and the second value showing the Euclidean distance only between the final point of simulated trajectory and the target. Continuous lines correspond to the hand trajectory, and dashed lines correspond to the target coordinate. **A**: Complete spinal circuitry model without perturbation. **B**: Complete spinal circuitry model with perturbation (increment of lower arm segment in 1 kg). **C**: Simpler spinal circuitry model (without spindle proprioception – only Renshaw pathway) without perturbation. **D**: Simpler spinal circuitry model with perturbation. For reference, the evaluated target is indicated in the bottom.

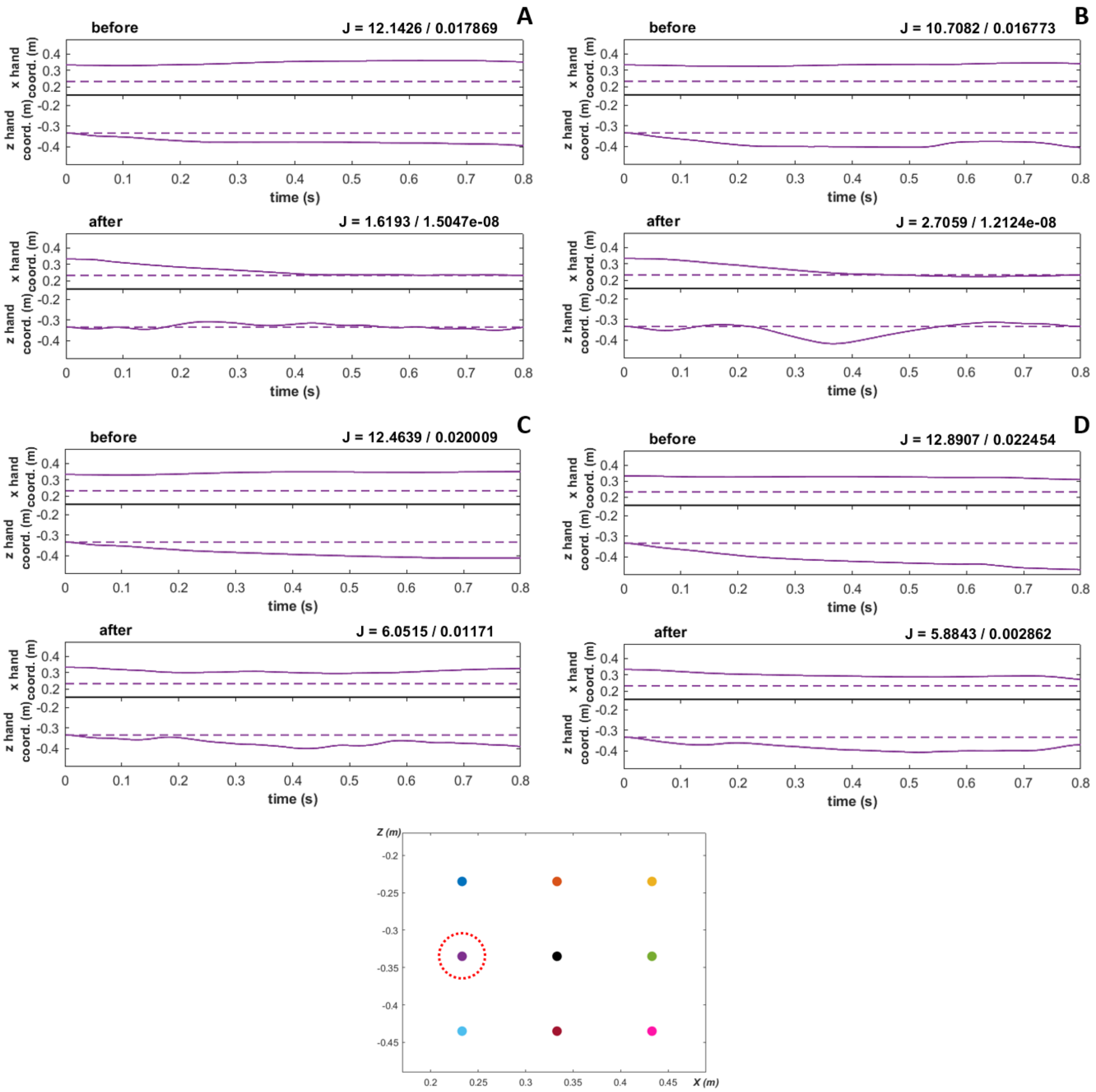

Figure S4. Reaching hand-trajectory per coordinate versus time – target $T4$ ([0.23,-0.33]m). Before completion of optimization (first optimization iteration) and after completion of optimization (best solution found). J corresponds to the value of cost functions used during optimization, with the first value representing the sum of Euclidean distances between all points of the simulated trajectory to the target, and the second value showing the Euclidean distance only between the final point of simulated trajectory and the target. Continuous lines correspond to the hand trajectory, and dashed lines correspond to the target coordinate. **A**: Complete spinal circuitry model without perturbation. **B**: Complete spinal circuitry model with perturbation (increment of lower arm segment in 1 kg). **C**: Simpler spinal circuitry model (without spindle proprioception – only Renshaw pathway) without perturbation. **D**: Simpler spinal circuitry model with perturbation. For reference, the evaluated target is indicated in the bottom.

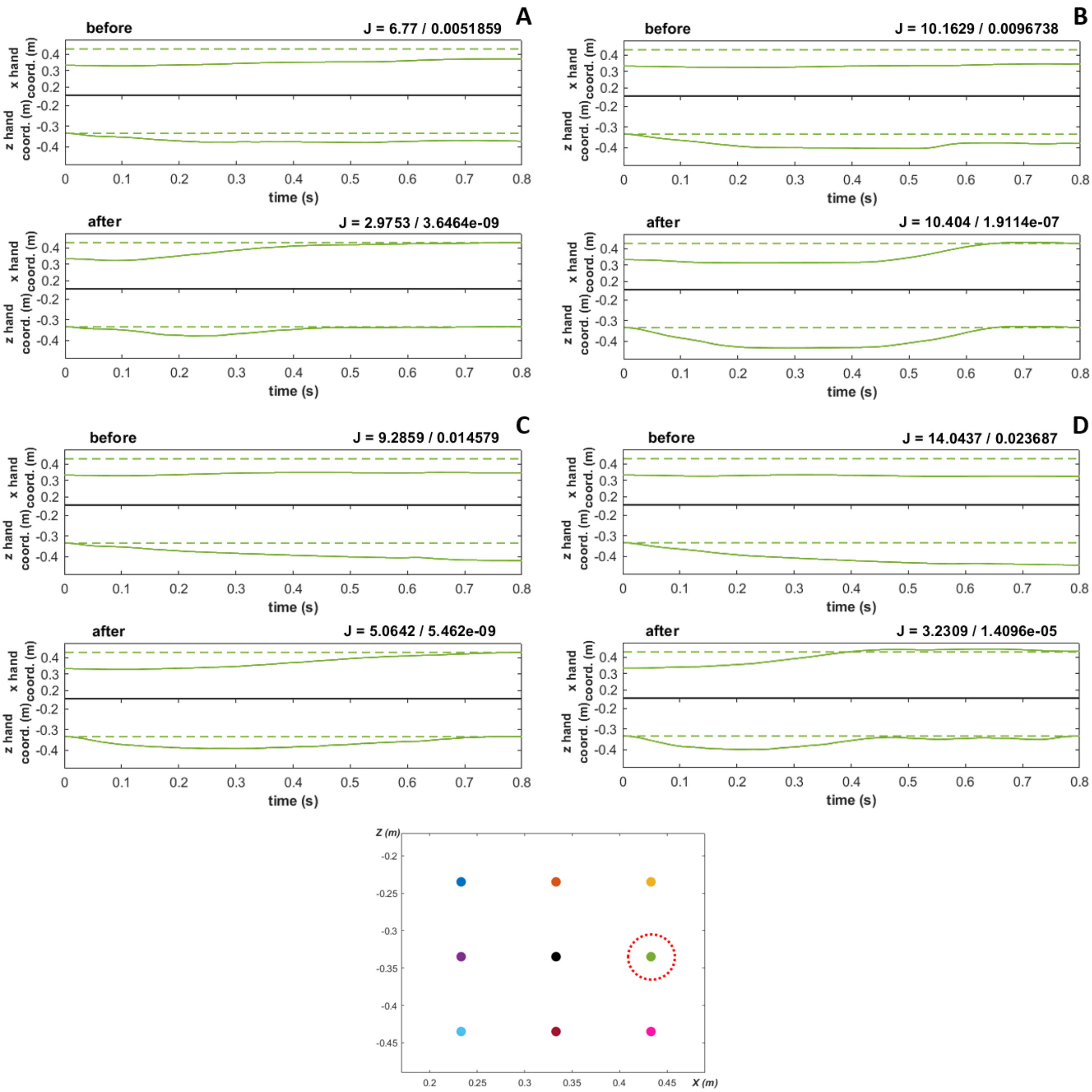

Figure S5. Reaching hand-trajectory per coordinate versus time – target $T5$ ([0.43,-0.33]m). Before completion of optimization (first optimization iteration) and after completion of optimization (best solution found). J corresponds to the value of cost functions used during optimization, with the first value representing the sum of Euclidean distances between all points of the simulated trajectory to the target, and the second value showing the Euclidean distance only between the final point of simulated trajectory and the target. Continuous lines correspond to the hand trajectory, and dashed lines correspond to the target coordinate. **A**: Complete spinal circuitry model without perturbation. **B**: Complete spinal circuitry model with perturbation (increment of lower arm segment in 1 kg). **C**: Simpler spinal circuitry model (without spindle proprioception – only Renshaw pathway) without perturbation. **D**: Simpler spinal circuitry model with perturbation. For reference, the evaluated target is indicated in the bottom.

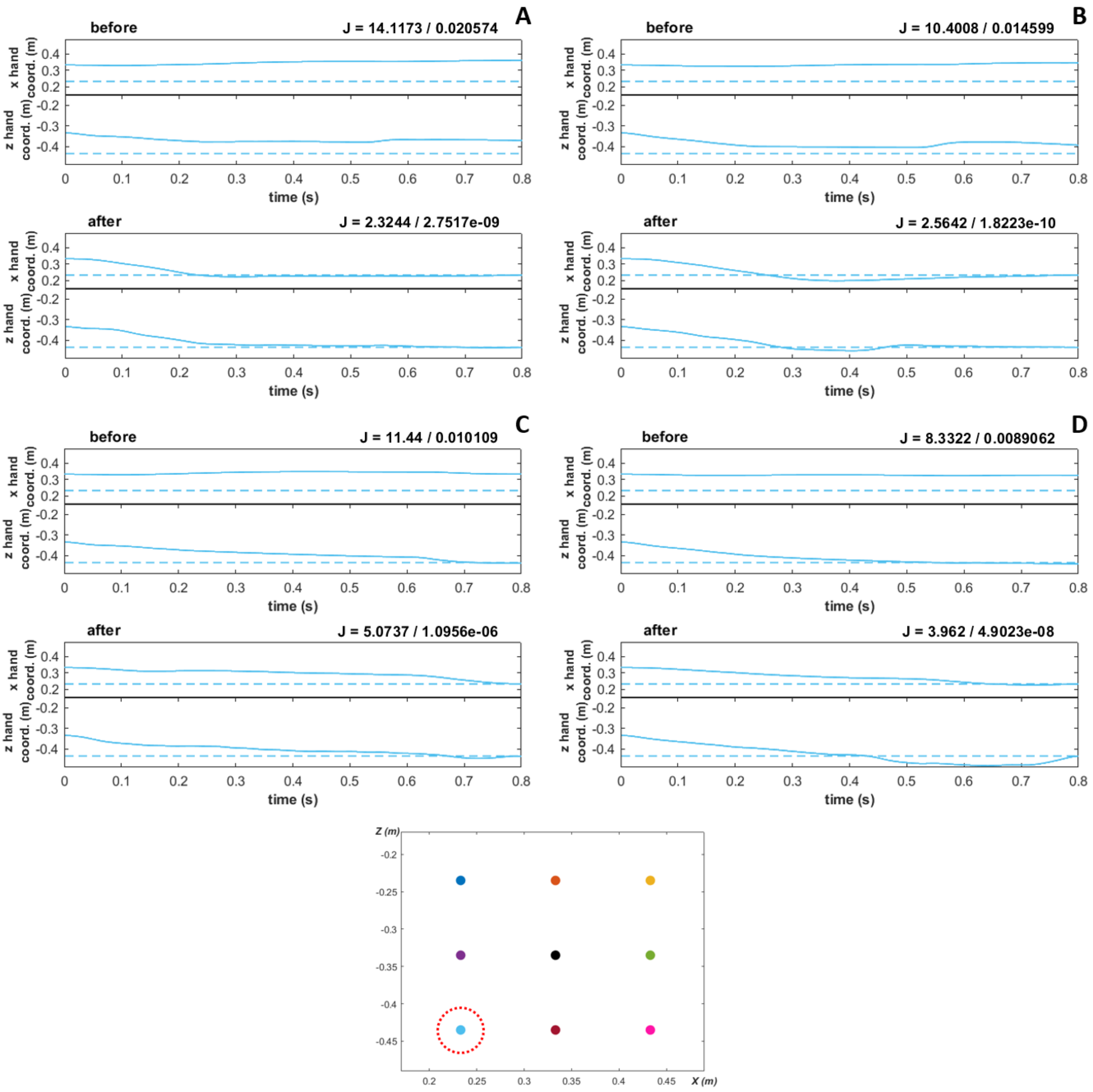

Figure S6. Reaching hand-trajectory per coordinate versus time – target $T6$ ([0.23,-0.43]m). Before completion of optimization (first optimization iteration) and after completion of optimization (best solution found). J corresponds to the value of cost functions used during optimization, with the first value representing the sum of Euclidean distances between all points of the simulated trajectory to the target, and the second value showing the Euclidean distance only between the final point of simulated trajectory and the target. Continuous lines correspond to the hand trajectory, and dashed lines correspond to the target coordinate. **A**: Complete spinal circuitry model without perturbation. **B**: Complete spinal circuitry model with perturbation (increment of lower arm segment in 1 kg). **C**: Simpler spinal circuitry model (without spindle proprioception – only Renshaw pathway) without perturbation. **D**: Simpler spinal circuitry model with perturbation. For reference, the evaluated target is indicated in the bottom.

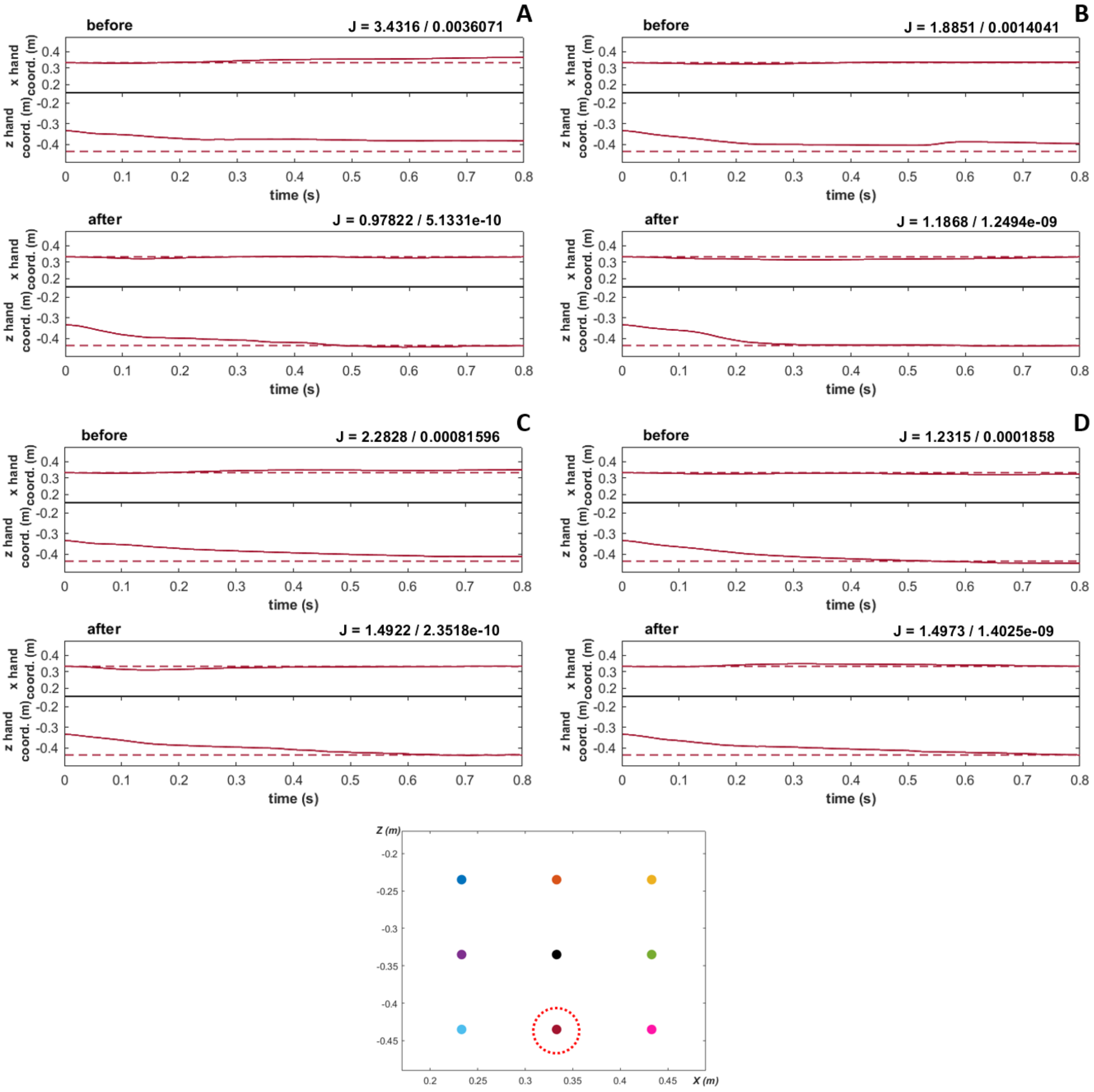

Figure S7. Reaching hand-trajectory per coordinate versus time – target $T7$ ([0.33,-0.43]m). Before completion of optimization (first optimization iteration) and after completion of optimization (best solution found). J corresponds to the value of cost functions used during optimization, with the first value representing the sum of Euclidean distances between all points of the simulated trajectory to the target, and the second value showing the Euclidean distance only between the final point of simulated trajectory and the target. Continuous lines correspond to the hand trajectory, and dashed lines correspond to the target coordinate. **A**: Complete spinal circuitry model without perturbation. **B**: Complete spinal circuitry model with perturbation (increment of lower arm segment in 1 kg). **C**: Simpler spinal circuitry model (without spindle proprioception – only Renshaw pathway) without perturbation. **D**: Simpler spinal circuitry model with perturbation. For reference, the evaluated target is indicated in the bottom.

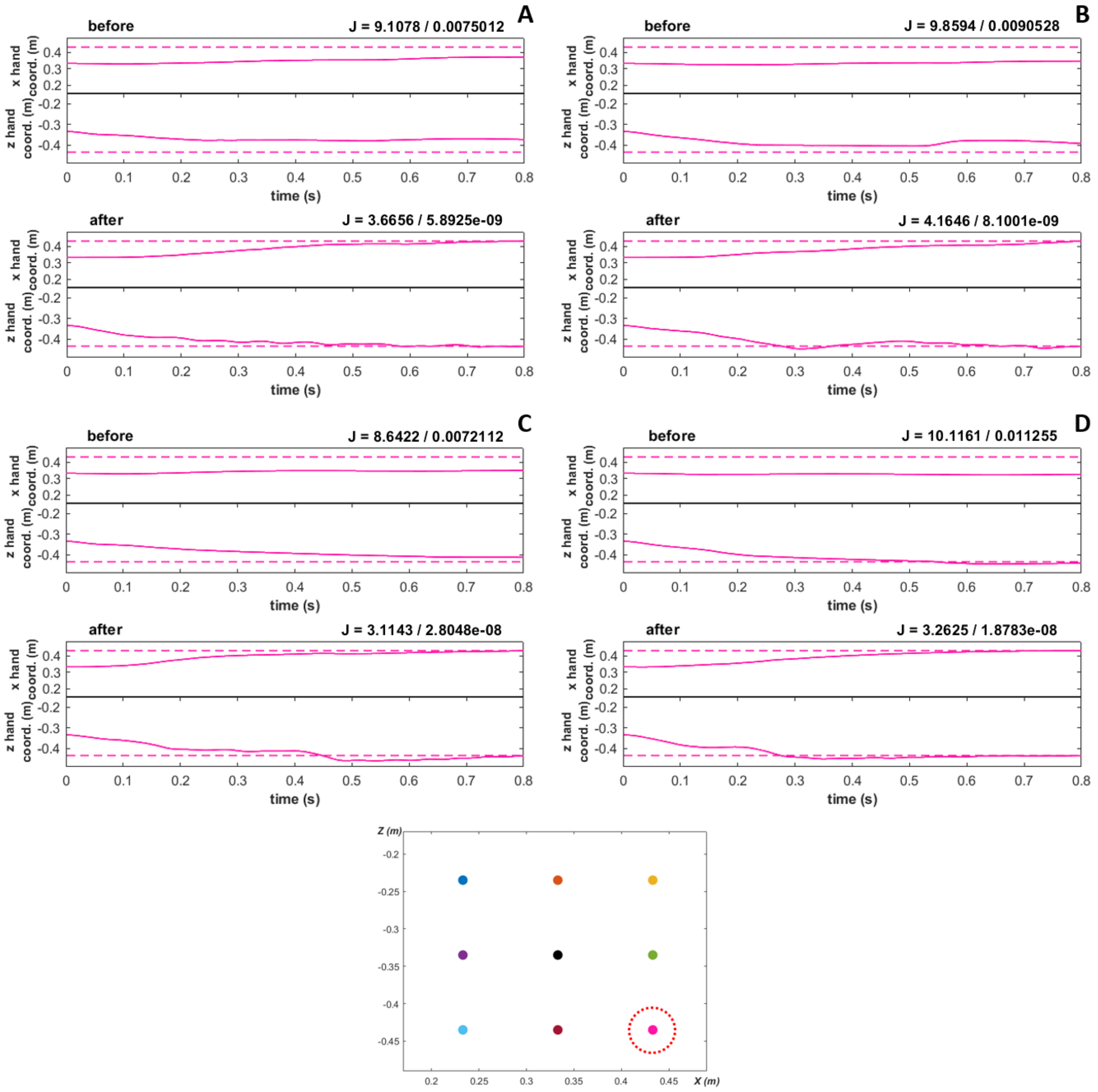

Figure S8. Reaching hand-trajectory per coordinate versus time – target $T8$ ([0.43,-0.43]m). Before completion of optimization (first optimization iteration) and after completion of optimization (best solution found). J corresponds to the value of cost functions used during optimization, with the first value representing the sum of Euclidean distances between all points of the simulated trajectory to the target, and the second value showing the Euclidean distance only between the final point of simulated trajectory and the target. Continuous lines correspond to the hand trajectory, and dashed lines correspond to the target coordinate. **A**: Complete spinal circuitry model without perturbation. **B**: Complete spinal circuitry model with perturbation (increment of lower arm segment in 1 kg). **C**: Simpler spinal circuitry model (without spindle proprioception – only Renshaw pathway) without perturbation. **D**: Simpler spinal circuitry model with perturbation. For reference, the evaluated target is indicated in the bottom.

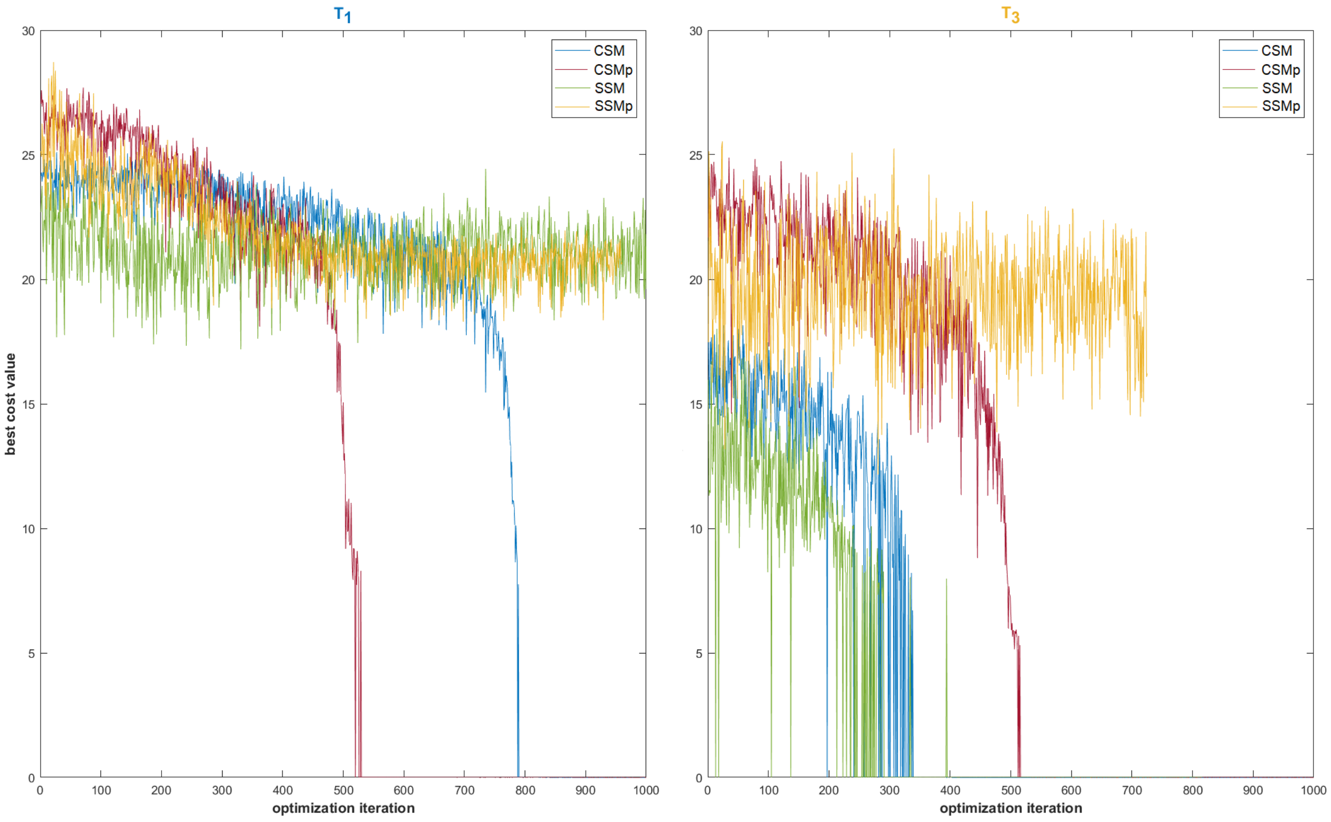

Figure S9. Learning curves for the targets $T1$ ([0.23,-0.23]m) and $T3$ ([0.43,-0.23]m). It is shown how the best cost value develops over the optimization iterations of the CMA-ES algorithm for the four evaluated scenarios. **CSM**: Complete spinal circuitry model without perturbation. **CSMp**: Complete spinal circuitry model with perturbation (increment of lower arm segment in 1 kg). **SSM**: Simpler spinal circuitry model (without spindle proprioception – only Renshaw pathway) without perturbation. **SSMp**: Simpler spinal circuitry model with perturbation.

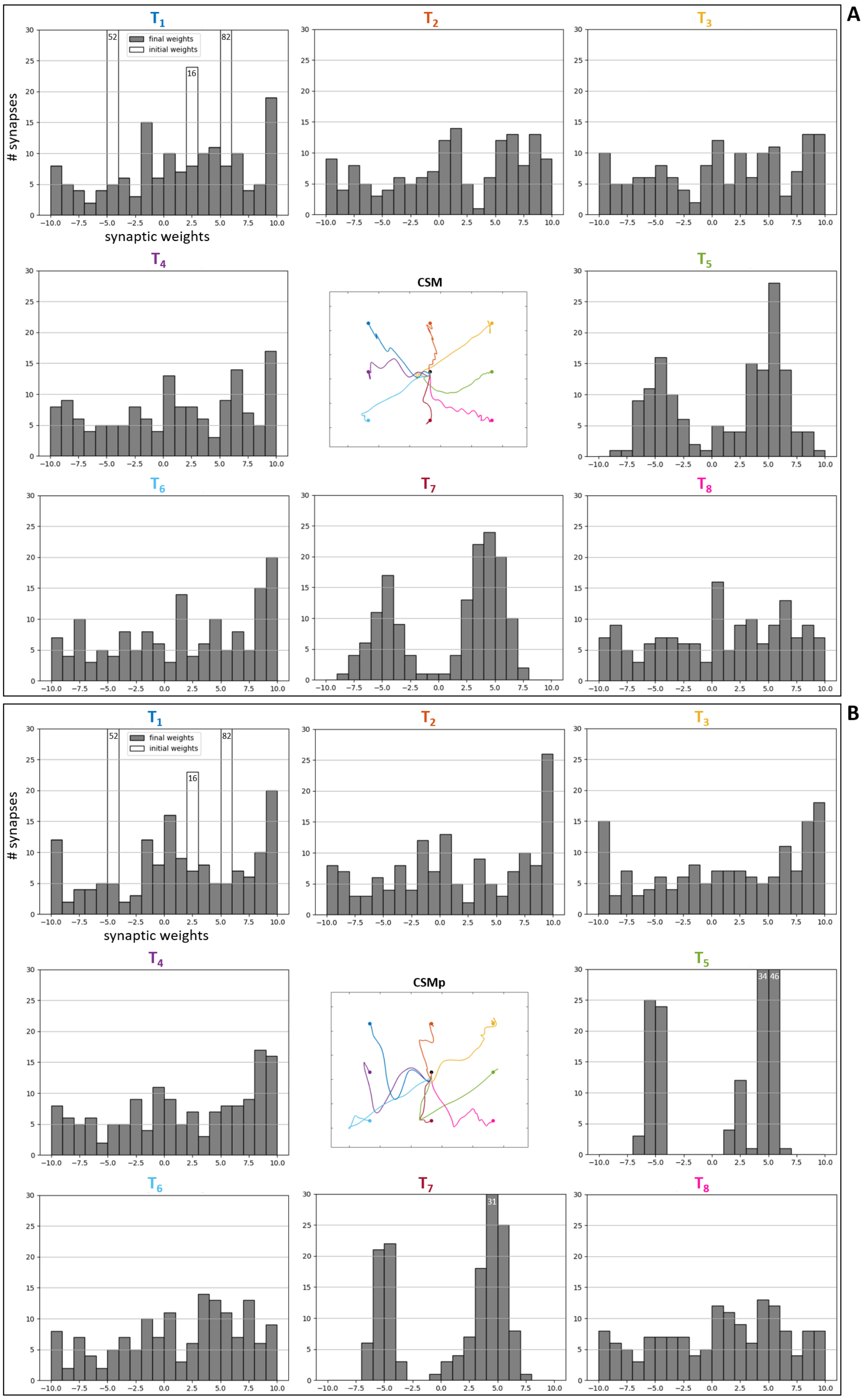

Figure S10. Histograms of final synaptic weights for all neuronal connections in the complete spinal circuitry model. **A**: Without perturbation. **B**: With perturbation. The final weight values from the individual optimizations for the eight evaluated targets ($T1$ to $T8$) are presented as histograms, according to the targets' position, together with a reduced version of the resulting trajectories for reference. For comparison, the initial weight values are also included in the histogram of the first target, corresponding to the same initial weights in all targets' optimizations.

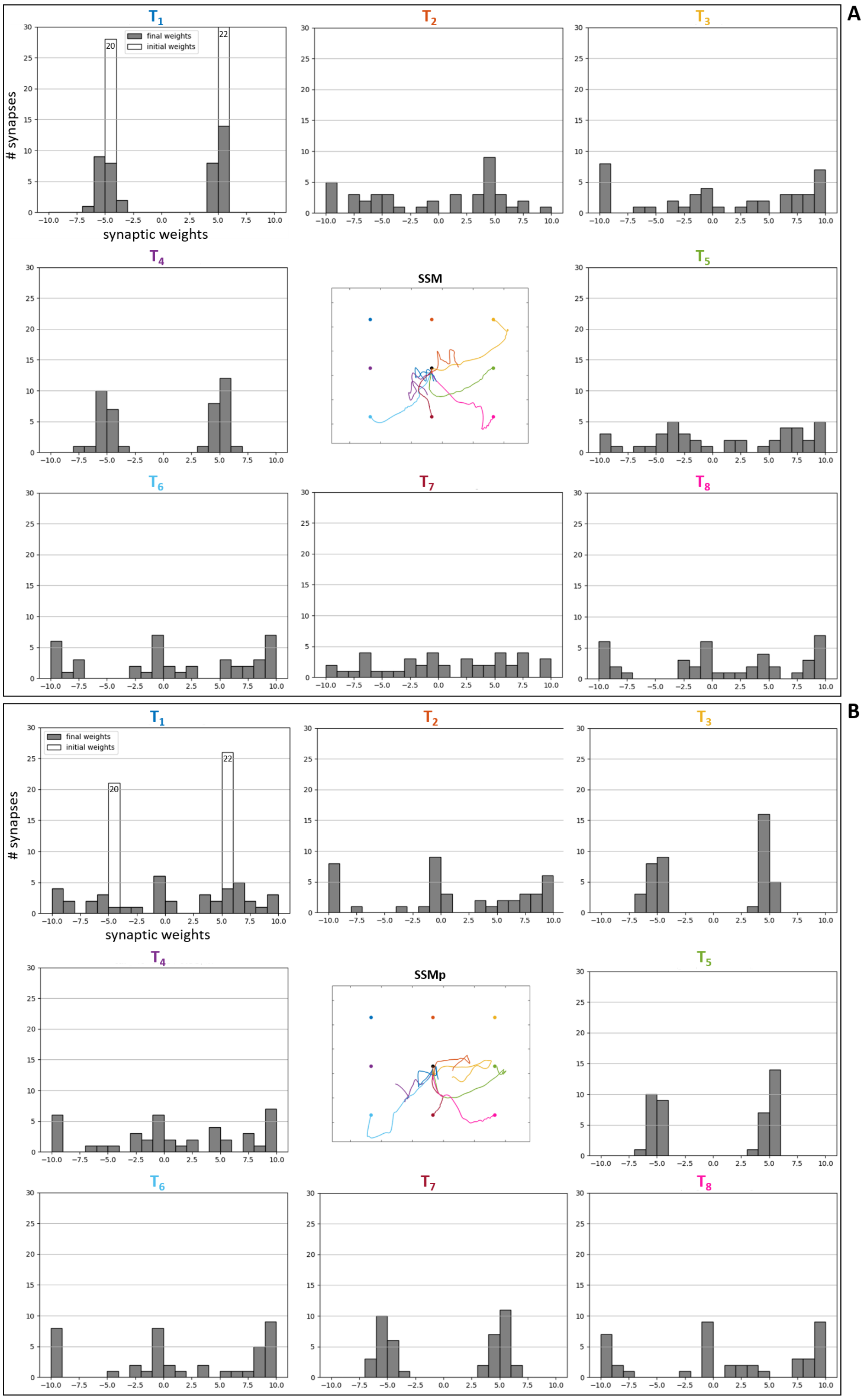

Figure S11. Histograms of final synaptic weights for all neuronal connections in the simpler spinal circuitry model. **A**: Without perturbation. **B**: With perturbation. The final weight values from the individual optimizations for the eight evaluated targets ($T1$ to $T8$) are presented as histograms, according to the targets' position, together with a reduced version of the resulting trajectories for reference. For comparison, the initial weight values are also included in the histogram of the first target, corresponding to the same initial weights in all targets' optimizations.
